# Recombinant SARS-CoV-2 envelope protein traffics to the *trans*-Golgi network following amphipol-mediated delivery into human cells

**DOI:** 10.1016/j.jbc.2021.100940

**Published:** 2021-07-05

**Authors:** James M. Hutchison, Ricardo Capone, Dustin D. Luu, Karan H. Shah, Arina Hadziselimovic, Wade D. Van Horn, Charles R. Sanders

**Affiliations:** 1 Chemical and Physical Biology Graduate Program, Vanderbilt University, Nashville, Tennessee, USA; 2 Center for Structural Biology, Vanderbilt University, Nashville, Tennessee, USA; 3 Department of Biochemistry, Vanderbilt University, Nashville, Tennessee, USA; 4 School of Molecular Sciences, Arizona State University, Tempe, Arizona, USA; 5 The Biodesign Institute Centers for Personalized Diagnostics and Mechanisms of Evolution, Arizona State University, Tempe, Arizona, USA; 6 Department of Medicine, Vanderbilt University Medical Center, Nashville, Tennessee, USA

**Keywords:** SARS, coronavirus, envelope, E protein, amphipol, ion channel, lysosomes, membrane, insertion, *trans*-Golgi network, AF488, Alexa Flour 488, CoV, coronavirus, ER, endoplasmic reticulum, ERGIC, ER–Golgi complex intermediate compartment, LAMP1, lysosomal-associated membrane protein 1, NBD, nitrobenzoxadiazole, NIH, National Institutes of Health, S2-E, SARS-CoV-2 envelope protein, S2-E-NBD, NBD-labeled S2-E, SARS-CoV-2, severe acute respiratory syndrome coronavirus 2, TGN, *trans*-Golgi network, WGA, wheat germ agglutinin

## Abstract

The severe acute respiratory syndrome coronavirus 2 envelope protein (S2-E) is a conserved membrane protein that is important for coronavirus (CoV) assembly and budding. Here, we describe the recombinant expression and purification of S2-E in amphipol-class amphipathic polymer solutions, which solubilize and stabilize membrane proteins, but do not disrupt membranes. We found that amphipol delivery of S2-E to preformed planar bilayers results in spontaneous membrane integration and formation of viroporin cation channels. Amphipol delivery of the S2-E protein to human cells results in plasma membrane integration, followed by retrograde trafficking to the *trans*-Golgi network and accumulation in swollen perinuclear lysosomal-associated membrane protein 1–positive vesicles, likely lysosomes. CoV envelope proteins have previously been proposed to manipulate the luminal pH of the *trans*-Golgi network, which serves as an accumulation station for progeny CoV particles prior to cellular egress *via* lysosomes. Delivery of S2-E to cells will enable chemical biological approaches for future studies of severe acute respiratory syndrome coronavirus 2 pathogenesis and possibly even development of “Trojan horse” antiviral therapies. Finally, this work also establishes a paradigm for amphipol-mediated delivery of membrane proteins to cells.

The severe acute respiratory syndrome coronavirus 2 (SARS-CoV-2) became a focal point of science and society in 2020. It is to be hoped that the ongoing vaccine development and delivery programs will soon allow the world to return to an approximation of normalcy (1, 2). However, previous coronavirus (CoV) epidemics, including Middle East respiratory syndrome (3) and SARS (4), from 2002 to 2003 foretell that future CoV zoonotic events (5) are likely to afflict humankind. Fundamental studies of the molecular underpinnings of CoVs may help to mitigate the current and future pandemics.

Within CoVs, there are four critically conserved structural proteins (6, 7), each of which is of possible therapeutic importance because of their critical functions (8). Among these is the envelope (E) protein. The E protein is a single-pass transmembrane protein whose roles in pathogenesis are incompletely understood (9). However, its importance is highlighted by cellular studies showing that the CoV E and M proteins alone are sufficient to produce a budding virus-like particle (10, 11, 12). Moreover, deletion of E dramatically lowers viral fitness (13, 14, 15), and growing evidence suggests that E is directly responsible for acute respiratory distress syndrome occurring in conjunction with CoV infections (16, 17). E is highly expressed in infected cells, but only a small fraction is incorporated into mature viral particles, implying functions beyond its role as a mature capsid structural protein (18). Supporting this idea, E is known to populate both monomer and oligomer forms *in vivo* (19). Most biophysical measurements have focused on the homopentamer form that functions as a cation-selective ion channel (20, 21, 22, 23), which is analogous to a well-studied and validated drug target, the influenza M2 protein (24, 25).

A distinct feature of CoV assembly is that its nascent particles (26) accumulate in the *trans*-Golgi network (TGN) (27) before undergoing a unique cellular egress pathway *via* deacidified lysosomes (28). The E protein is critical to viral maturation (10, 18, 29). Localization of SARS E protein to these membranes is remarkably stringent, likely a consequence of Golgi-targeting motifs present in the E protein (29). Since E functions in multiple roles that are critical to viral fitness (30, 31, 32), it is desirable to develop methods to further characterize key E-dependent pathogenic mechanisms. Current methods to study the E protein in mammalian cells are reliant on transfection of genetic material encoding the protein into cells and its subsequent transcription and translation. Here, we sought to develop a robust method for exogenous delivery of purified SARS-CoV-2 envelope protein (S2-E) into human cell lines to enable chemical biological methods for studies of S2-E function and possibly to potentiate novel therapeutic approaches.

## Results and discussion

We developed a straightforward bacterial expression and purification protocol that yields ∼100 μg per liter of culture of 90 to 95% pure full-length S2-E under conditions in which it is bereft of detergent and lipid, with its aqueous solubility being maintained by complexation with the zwitterionic amphipol poly (maleic anhydride-alt-1-decene) substituted with 3-(dimethylamino) propylamine (33, 34) (Fig. S1 and Supporting Information [Materials and Methods] section). This purification protocol has been streamlined to a single gravity column and does not require semiautomated chromatography or an ultracentrifuge. Once purified into lipid/detergent-free amphipol solution, the S2-E–amphipol complexes remain stable and soluble in aqueous solution even following removal of excess uncomplexed amphipols. Amphipols are a class of amphipathic polymers that exhibit weak detergent properties, in that they can solubilize and stabilize membrane proteins but cannot solubilize or even permeabilize membranes (35, 36). In addition, some amphipols are well tolerated by animals (37) and have been used in vaccine development (36, 38) because at least some forms do not elicit the production of anti-amphipol antibodies (39).

**Figure 1:**
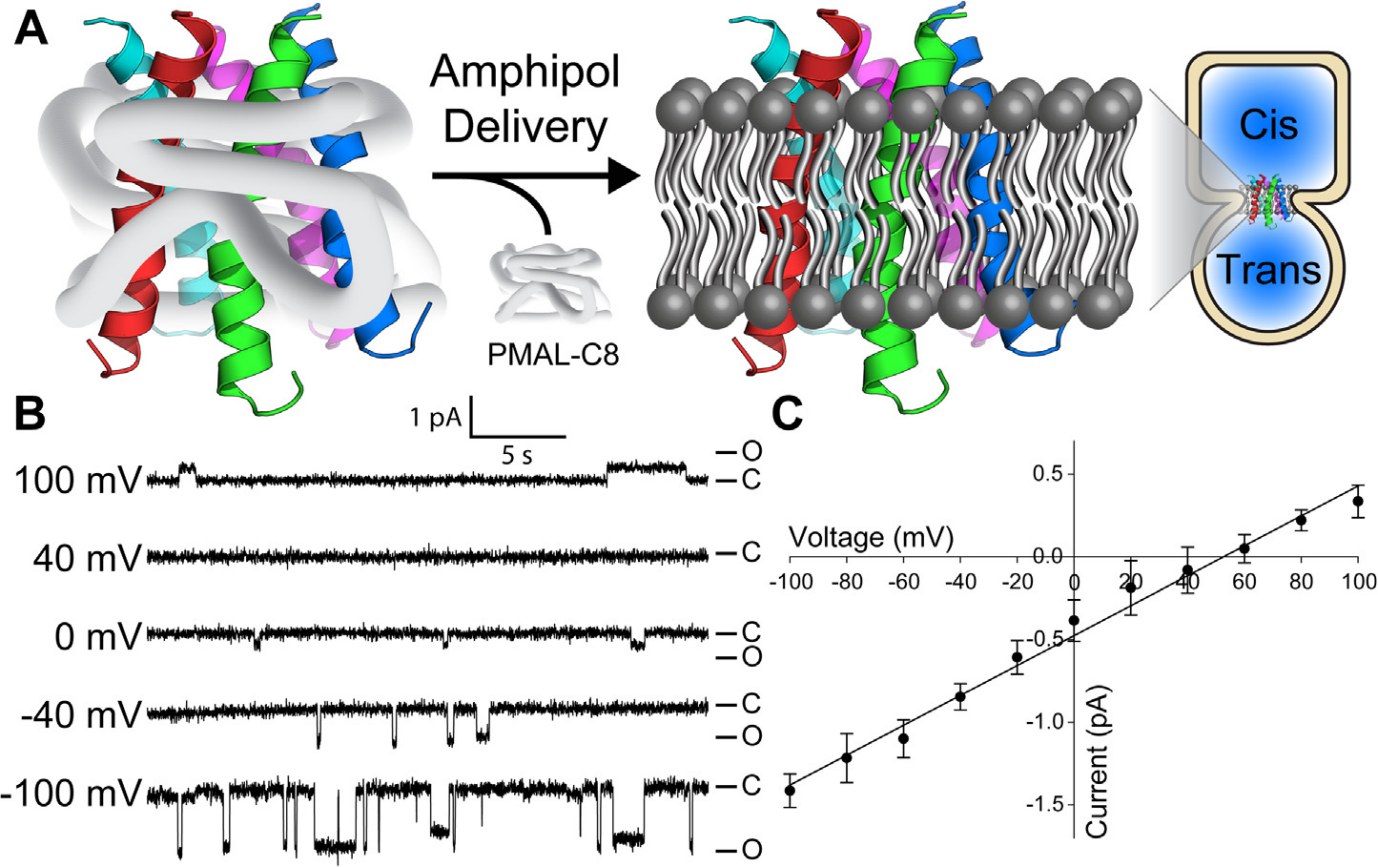
**Functional delivery of SARS-CoV-2 envelope protein from amphipol complexes to planar lipid bilayers.***A*, schematic of SARS-CoV-2 envelope protein (S2-E) delivered using amphipols for membrane protein insertion into planar lipid bilayers. *B*, representative single-channel current recordings of PMAL-C8 amphipol-delivered S2-E as a function of transmembrane electrical potential showing ion channel activity in POPC:POPE (3:1) planar bilayers, where S2-E fluctuates between closed (C) and open (O) states. *C*, the S2-E current–voltage relationship identifies a conductance of 9.0 ± 0.3 pS and a reversal potential of 53 ± 3 mV in an asymmetric NaCl buffer, indicative of cation selectivity. Data represent three replicates. Error bars represent standard deviation from the three distinct amphipol delivery experiments on different days (for open state probabilities, see Fig. S2). Single-channel current recordings using S2-E-NBD also show similar conductance (Fig. S3). The *trans* compartment is the chamber with the command/input Ag–AgCl electrode, whereas the grounded chamber is the *cis* compartment. Planar bilayer control experiments in which PMAL-C8 only was added (no S2-E) did not exhibit channel activity at amphipol concentrations 1× or 50× relative to those used in these S2-E and S2-E-NBD planar bilayer experiments. PMAL-C8, poly (maleic anhydride-alt-1-decene) substituted with 3-(dimethylamino) propylamine; POPC, 1-palmitoyl-2-oleoyl-sn-glycero-3-phosphocholine; POPE, 1-palmitoyl-2-oleoyl-sn-glycero-3-phosphoethanolamine; S2-E-NBD, nitrobenzoxadiazole–labeled S2-E; SARS-CoV-2, severe acute respiratory syndrome coronavirus 2.

Planar lipid bilayer electrophysiology was used to test if amphipols can deliver the S2-E protein to a membrane environment to form ion channels without otherwise disrupting the lipid bilayer (Fig. 1*A*). It was seen that amphipol-based S2-E delivery resulted in ion channel activity that is consistent with previous SARS-CoV-1E (40) and preliminary S2-E (17) channel measurements in terms of current amplitudes, sodium cation selectivity, and open probabilities (Fig. 1, *B* and *C* and Fig. S2 and Supporting Information [Materials and Methods] section). The S2-E–dependent currents and similarity to other planar bilayer measurements support the idea that S2-E is released spontaneously from amphipols into membranes. The bilayer integrity during amphipol delivery and exposure was monitored through membrane capacitance measurements. The bilayers remained stable throughout the recordings with an average value of 58 ± 3 pF. These results demonstrate that recombinant S2-E can be delivered into preformed lipid bilayers using amphipols, where the protein not only inserts into the bilayers but also retains ion channel function, without compromising the bilayer integrity.

We next tested whether S2-E can be delivered from amphipol complexes to the membranes of human cells. To this end, S2-E was irreversibly tagged at one of its three native cysteine residues with fluorophores nitrobenzoxadiazole (NBD) or Alexa Flour 488 (AF488). The native cysteine residues do not form disulfide bonds *in vivo* (41) and are not required for ion channel formation (42, 43), although substituting two or three cysteines to alanine results in reduced viral fitness (41). NBD-labeling S2-E at roughly one cysteine site per protein did not significantly lower the channel open state probabilities (*p* values of 0.125 at −100 mV and 0.055 at 100 mV) and did not change channel conductance properties (Fig. S3) (44, 45). The solvatochromic properties of NBD (44) were better for observing the deposition of S2-E on the plasma membrane at early time points (Fig. S4) while the environment insensitivity of AF488 (45) was more suited for S2-E colocalization experiments. No significant difference was observed between S2-E labeled with either AF488 or NBD when quantifying colocalization (Fig. S5).

**Figure 2:**
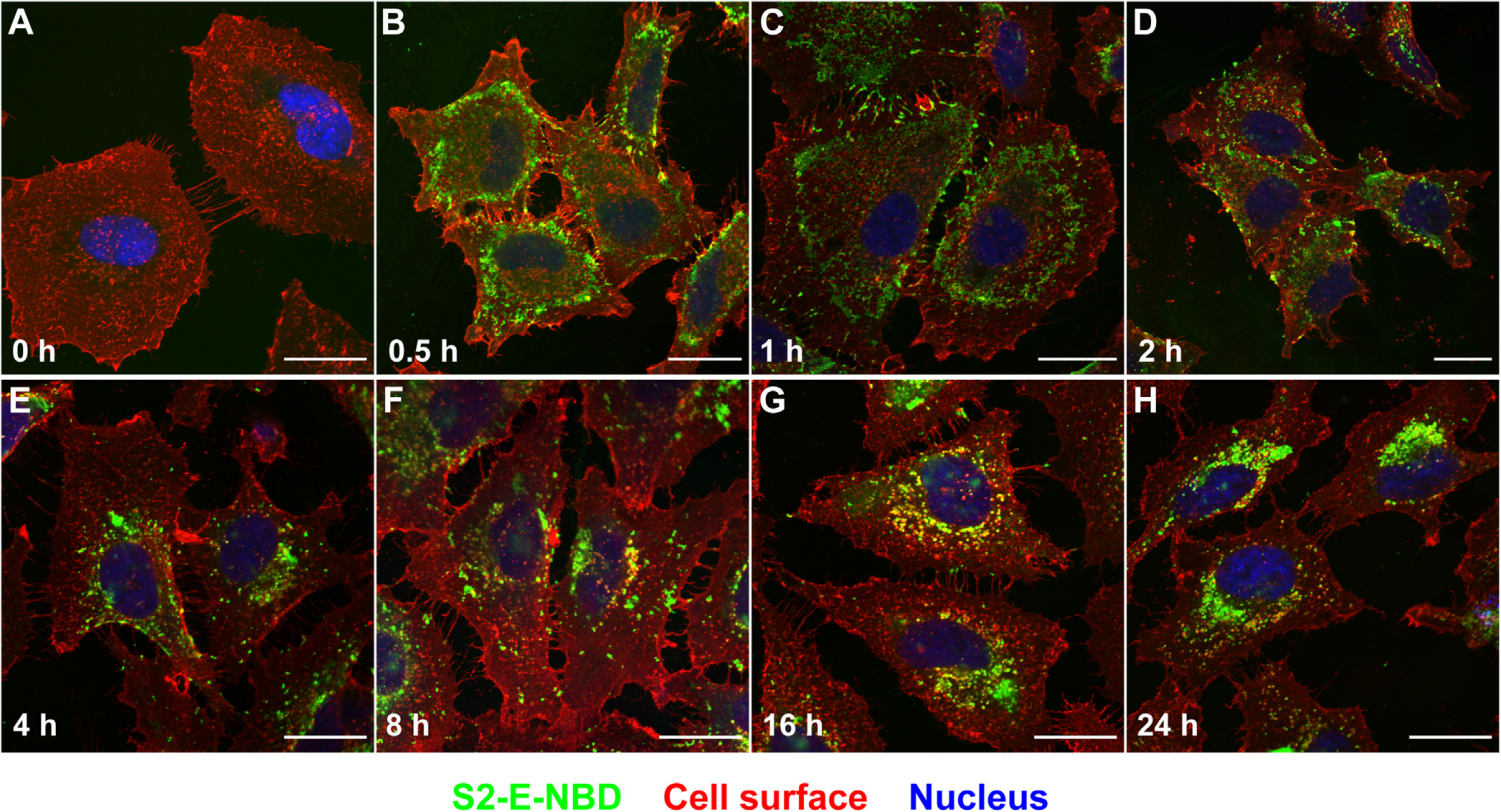
**Membrane incorporation and uptake of amphipol-delivered SARS CoV-2E protein by cells and subsequent intracellular trafficking of the protein.** Representative confocal microscopy images of HeLa cells at various time points following treatment with amphipol-complexed 2.5 μM S2-E labeled with NBD (S2-E-NBD). The following are the color markers: *green*, S2-E-NBD; *red*, cell membrane, for which we used WGA-AF555 (WGA is an *N*-acetylglucosamine and sialic acid–binding lectin, here conjugated to AF555); and *blue*, cell nuclei, for which we used 1,5-bis((2-(di-methylamino) ethyl)amino)-4, 8-dihydroxyanthracene-9,10-dione (a cell permeable, far-red emitting fluorescent anthraquinone dye, that stains dsDNA). (*A*) is the untreated (0 μM) sample and 0 h time point, (*B*) is cells 0.5 h after treatment, (*C*) is following 1 h, (*D*) 2 h, (*E*) 4 h, (*F*) 8 h, (*G*) 16 h, and (*H*) 24 h. The S2-E-NBD signal migrates from the cell plasma membrane (see panel *B*) toward the perinuclear space (see panels *G* and *H*). Time-course experiments using the same cell markers were independently repeated three times using three different S2-E-NBD preparations. All scale bars represent 25 μm. See further details in the Supporting Information (Materials and Methods) section and Fig. S6. SARS CoV-2 E, severe acute respiratory syndrome coronavirus 2 envelope protein; WGA, wheat germ agglutinin.

As shown in Figure 2 and Fig. S6, the NBD-labeled S2-E (S2-E-NBD) protein was delivered from amphipol complexes to HeLa cell membranes, with all cells exhibiting NBD signal within 30 min (Fig. 2*B*). Figure 2, *C*–*F* shows the 8 h progression of the S2-E-NBD protein from the plasma membrane to a predominately perinuclear intracellular location. After 16 to 18 h, nearly all the S2-E proteins were observed in the vicinity of the nucleus, with a clear focal area on one side of the nuclear compartment (Fig. 2, *G* and *H*). Delivered S2-E was typically more diffuse at early time points but becomes punctate as it traffics toward the perinuclear space.

The amount of S2-E signal in cells was dependent on the applied amphipol/S2-E dose (Fig. S7). It was verified that NBD did not drive S2-E internalization and perinuclear aggregation (Fig. S7). To ensure that we were microscopically tracking intact S2-E-NBD instead of dye freed from full-length S2-E by degradation, we confirmed S2-E localization following cell fixation and permeabilization with a polyclonal anti-S2-E antibody (Fig. S5). We observed consistent strong colocalization between S2-E labeled with either AF488 or NBD and the polyclonal anti-E signal. Cysteine-reacted NBD was used as an S2-E degradation control and was found to stain all cellular membranes (Fig. S8). Therefore, delivered S2-E is not extensively degraded during the 24-h treatment since the level of anti-S2E and S2-E-dye colocalization remained strong at both short and long time points (Fig. S5). We observed unspecific binding of the anti-E antibody in the nucleus and determined the dye-tagged S2-E to be a better reporter of S2-E localization (Fig. S5). While amphipols have previously been reported to deliver select membrane proteins to artificial lipid bilayers (33, 46), this study represents the first use of amphipols to deliver a protein to mammalian cells. Elucidation of the pathway(s) taken by the S2-E protein to dissociate from its soluble amphipol complex to then insert into the membrane and adopt a transbilayer configuration will require further study.

To ensure that the observed S2-E delivery and perinuclear trafficking was not unique to HeLa cells, we also examined possible delivery of S2-E from amphipol solutions into SW1573 human alveolar cells, a coronavirus disease 2019–relevant cell line (47). We observed (Fig. S9) that S2-E is indeed taken up by these cells and similarly undergoes surface-to-perinuclear retrograde trafficking as seen in HeLa cells.

**Figure 3:**
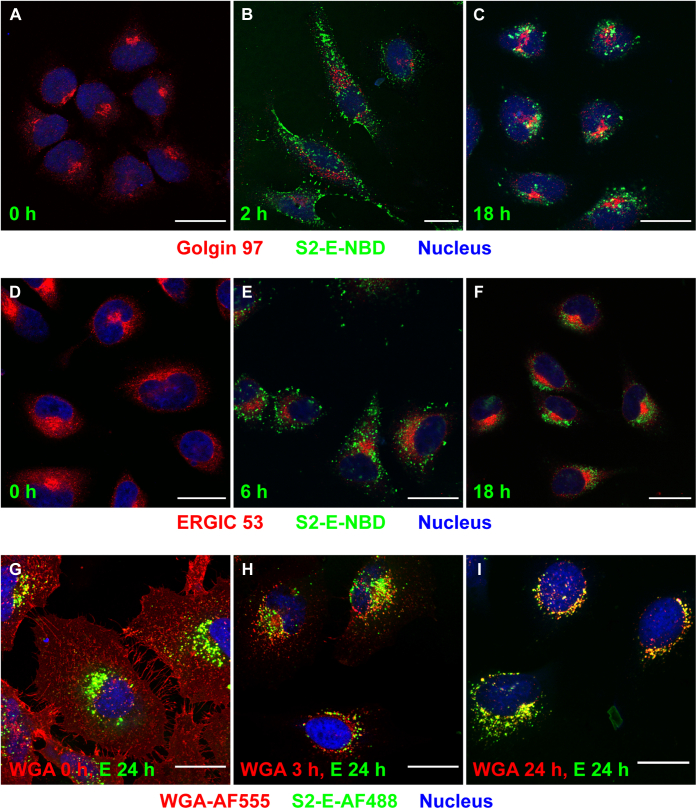
**SARS-CoV-2 envelope (S2-E) protein traffics to the perinuclear space and accumulates in the *trans*-Golgi network (TGN).** Representative confocal microscopy images showing HeLa cells treated with 2.5 μM S2-E labeled with either NBD or AF488. Panels *A*–*C* and *D*–*F* show S2-E localization relative to the Golgi (*A*–*C*) and ERGIC (*D*–*F*) as a function of increasing S2-E treatment time. Panels *G*–*I* show cells that were treated with S2-E-AF488 for 24 h and labeled with WGA-AF555, the latter added either (i) 24 h after S2-E-AF488 treatment followed by immediate fixation (*G*, 0 h of WGA), (ii) 21 h after S2-E-AF488 treatment followed by an additional 3 h of WGA incubation followed by fixation (*H*, 3 h of WGA), or (iii) at the same time (0 h), S2-E-AF488 was added to the cells followed by 24 h of incubation followed by fixation (*I*, 24 h of WGA). Notice how WGA accumulates and colocalizes with S2-E over time. Note also how WGA moves to the perinuclear space at a faster rate than S2-E; that is, compare 3 h WGA (*H*) *versus* S2-E at 2 h (*B*) or 6 h (*E*). Color scheme: *green*, S2-E labeled with NBD (*A*–*F*) or AF488 (*G*–*I*); *blue* is the fluorescent dye 1,5-bis((2-(di-methylamino) ethyl)amino)-4, 8-dihydroxyanthracene-9,10-dione, marking the cell nucleus; *red*, in panels *A*–*C*, is from an antibody to Golgin-97, a Golgi marker; in panels *D*–*F*, *red* is from an antibody to ERGIC-53, a defining marker for the ERGIC region and panels *F* and *G* is from WGA-AF555, a common marker for plasma membrane for fixed cells. Panels *A* and *D* are the control samples where cells were not treated with S2-E-NBD. Panels *A*–*I* were treated with labeled S2-E for the time indicated in *green fonts*, whereas WGA in panels *G*–*I* was added for the stated time shown in *red fonts*, right before cell fixation. Colocalization analysis of panel *I* between WGA-AF555 and S2-E-AF488 channels result in Pearson correlation coefficients (>0.7, Fig. S5). All experiments shown were repeated three times in panels *A*–*F* using three different E-NBD preparations, whereas in panels *G*–*I* twice with S2-E-NBD and once with S2-E-AF488. All three repeats show comparable behaviors. The scale bars represent 25 μm. Further details are provided in the Materials and Methods section of Supporting Information and Fig. S10. AF488, Alexa Flour 488; AF555, Alexa Flour 555; ERGIC, ER–Golgi complex intermediate compartment; NBD, nitrobenzoxadiazole; S2-E-AF488, AF488-labeled S2-E; SARS-CoV-2, severe acute respiratory syndrome coronavirus 2; WGA, wheat germ agglutinin.

The fact that delivered S2-E retrograde traffics proximal to one side of the nucleus (Fig. 2, *G* and *H*) is consistent with its localization at or near the Golgi compartments. To gain further insight into the final cellular location of S2-E, we used organelle-specific monoclonal antibodies and wheat germ agglutinin (WGA) to pinpoint the locations of the *cis*-Golgi and *trans*-Golgi, TGN, early endosomes, lysosomes, endoplasmic reticulum (ER), aggresomes, and ER–Golgi complex intermediate compartment (ERGIC) relative to delivered S2-E in the perinuclear space at later time points. In agreement with Figure 2, *B*–*E*, S2-E does not strongly colocalize with markers of ER–Golgi space at early time points and takes 18 to 24 h for the protein to accumulate in the perinuclear space *via* retrograde trafficking (Fig. 2, *G* and *H*). At 18 to 24 h after initial delivery, S2-E was not found in the ER, ERGIC, or *cis*-Golgi (Fig. 3, *D*–*F* and Figs. S10–S12). Instead, S2-E was seen to partially overlap with the *trans*-Golgi marker Golgin 97 (48) in the area surrounding the Golgi but not within the cis-Golgi (Fig. 3, *A*–*C* and Fig. S9).

The partial colocalization of S2-E with the *trans*-Golgi and exclusion from the *cis*-Golgi indicated that delivered S2-E ultimately resides in the TGN and associated vesicles. WGA is an *N*-acetylglucosamine and sialic acid-binding lectin (49, 50) that is often used in conjunction with fixed cells to stain the plasma membrane (51). In live cells, WGA is rapidly endocytosed to the Golgi and specifically accumulates in the endocytic TGN (Fig. S13) (52, 53, 54, 55). We observed strong colocalization between WGA and delivered S2-E between 16 and 24 h, indicating that S2-E accumulates in the TGN (Fig. 3, *G*–*I* and Fig. S10). WGA and S2-E travel to the TGN independently, and WGA does not induce S2-E TGN colocalization. There is significant bidirectional traffic between the TGN and endosome–lysosome systems (56), and we wanted to determine if delivered S2-E was accumulating in endosomes and lysosomes at later time points. We did observe some colocalization of S2-E with early endosome marker, early endosome antigen 1 (57), at later time points (Fig. S14). In addition, we observed colocalization of S2-E with late endosomes and lysosomes as indicated by the overlap in S2-E and lysosomal-associated membrane protein 1 (LAMP1) signals (Fig. S15) (58). Perinuclear S2-E containing LAMP1-positive vesicles were enlarged, whereas peripheral LAMP1-positive vesicles remained unchanged.

The localization of delivered S2-E to swollen TGN vesicles is likely disease relevant given that progeny CoVs are known to accumulate in the TGN before cellular egress (27, 59). Lysosomes have long been associated with CoV infection (60), and recent work showed that CoV is unique in its use of deacidified lysosomes for cellular egress (28). In line with these ideas, E is known to increase the pH of the Golgi (59) and TGN (61), apparently to protect the spike protein from premature processing. E also has been shown to slow cellular secretory pathways (62).

It is likely that the Golgi-localization motifs (29) in S2-E drive its retrograde trafficking in a way closely related to the mechanism that facilitates E protein perinuclear retention during viral infection. However, we also considered the possibility that amphipol-mediated extracellular delivery of S2-E triggers ER stress and unfolded protein response–related retrograde trafficking, leading to deposition of S2-E in perinuclear aggresomes. Aggresomes are ordered protein aggregates that form following transport of certain proteins along microtubules by dynein to perinuclear microtubule-organizing centers (63) and are often detected by the formation of vimentin cages (63). We looked but did not observe the induction of vimentin cages with our delivered S2-E (Fig. S16). The lack of aggresome formation suggests that our delivered S2-E does not induce an obvious unfolded protein response stress, likely because the S2-E accumulates in the TGN and not in the ER (64, 65, 66).

We did not observe an obvious enrichment of S2-E in the ERGIC or ER compartments before the accumulation in the TGN. However, it is important to note that we focused on identifying the final location of externally delivered S2-E and not its location in earlier time points. In addition, further studies will be required to understand by which pathways S2-E is trafficked from the plasma membrane to the TGN.

## Conclusions

**Figure 4:**
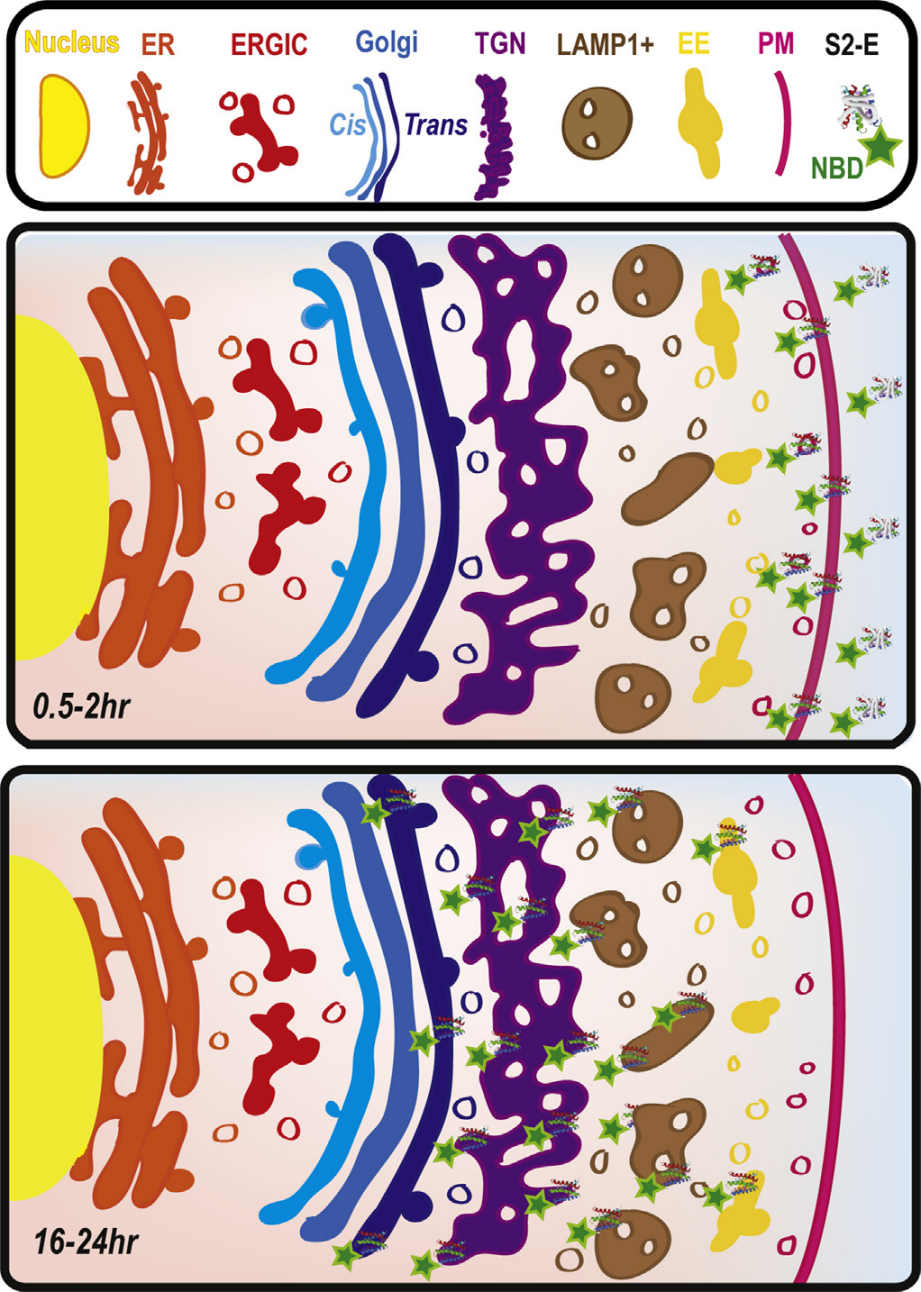
**Schematic of amphipol-delivered S2-E and its localization within HeLa cells at early and late time points.***Top panel*, legend of cellular markers tested for colocalization with S2-E. *Middle panel*, S2-E is mainly localized on the plasma membrane at early time points. *Bottom panel*, after 16 to 24 h, delivered S2-E is enriched within the TGN and LAMP1-positive vesicles. S2-E is also partially found within early endosomes and the *trans*-Golgi. Organelles and S2-E are not to scale. LAMP1, lysosomal-associated membrane protein 1; S2-E, severe acute respiratory syndrome coronavirus 2 envelope protein; TGN, *trans*-Golgi network.

We have shown that the S2-E protein can be stripped of lipid and detergent and purified into aqueous solutions in which its solubility is maintained solely by complexation with amphipols. The protein can then be delivered to lipid bilayers, in which the protein spontaneously inserts into the membrane to form ion channels. As proposed in Figure 4, addition of the S2-E protein to living human cells results in plasma membrane integration and subsequent retrograde trafficking deep within the cell, ultimately occupying enlarged LAMP1-positive vesicles derived from the TGN, which are believed to be critical for CoV egress and pH dysregulation.

The S2-E protein-to-cells approach established by this work should be exploitable as a route to delivering chemically modified full-length S2-E to cells in culture or possibly even to cells under physiological conditions. This capability enables a wide range of chemical biological tools to explore the biological function of this protein and test whether chemical warhead–armed S2-E can play the role of a Trojan horse to interfere with SARS-CoV-2 replication, potentially as an anti–COVID-19 therapeutic or prophylactic. The results of this work also establish a general paradigm for using amphipols to deliver membrane proteins to living cells, although whether numerous other membrane proteins can be successfully delivered using this approach remains to be explored.

## Data availability

All data needed to evaluate the conclusions mentioned in the article and supporting information are presented in this article or in the supporting information. Correspondence and requests for materials should be addressed to W. D. V. H. (wade.van.horn@asu.edu) or C. R. S. (chuck.sanders@vanderbilt.edu).

## Supporting information

This article contains supporting information.

## Conflict of interest

The authors declare that they have no conflicts of interest with the contents of this article.
