# Supplementary material for: Recombinant SARS-CoV-2 envelope protein traffics to the trans-Golgi network following amphipol-mediated delivery into human cells
Source: J Biol Chem. 2021 Jul 5;297(2):100940. doi: 10.1016/j.jbc.2021.100940 (PMC8256659; doi:10.1016/j.jbc.2021.100940)
Supplement: Supporting Materials and Methods and Figures S1–S16 [file mmc1.pdf]

Supporting Information for:

Recombinant SARS-CoV-2 envelope protein traffics to the trans-Golgi network following amphipol-mediated delivery into human cells

James M. Hutchison, Ricardo Capone, Dustin D. Luu, Karan H. Shah, Arina Hadziselimovic,

Wade D. Van Horn, and Charles R. Sanders

Contents

Supporting Materials and Methods

Supporting References

Supporting Figures S1-S16

Supporting Materials and Methods

Recombinant SARS-CoV-2 Envelope protein construct

DNA encoding the SARS CoV-2 envelope protein (S2-E) sequence followed by a C-terminal linker with a thrombin cleavage site and 10X His tag was inserted into a pET-21b plasmid vector. The encoded S2-E sequence is as shown below, with the added linker/thrombin site/tag indicated in blue font:

MYSFVSEETGTLIVNSVLLFLAFVVFLLVTLAILTALRLCAYCCNIVNVSLVKPSFYVYS-RVKNLNSSRVPDLLVLESSGGGSILVPRGSGGSHHHHHHHHHH

Multiple constructs were tested for expression in several bacterial strains and the inclusion of the linker with thrombin cut site significantly increased expression levels.

Recombinant expression of the SARS-CoV-2 Envelope protein

The expression plasmid indicated above was transformed into Rosetta 2(DE3) pLysS cells. Transformed cells were then spread on agar LB plates supplemented with ampicillin and chloramphenicol before overnight growth at 37 °C. Colonies were picked the next afternoon and used to inoculate LB medium (150 mL in 500 mL baffled flask) supplemented with 100 µM ampicillin and 34 µM chloramphenicol before culturing the bacteria overnight with rotary shaking at 37 °C at 230 rotations per minute (RPM). The next morning, 20 mL of overnight culture was added to 1 L of antibiotic-supplemented M9 medium with added MEM vitamins (Corning) in a 2 L baffled flask and grown at 37 °C at 230 RPM. After ~5-8

hours, when the cultures had grown to an OD_{600} of 0.6-0.8 they were induced with 1 mM isopropyl β-D-1-thiogalactopyranoside. Induced cultures were allowed to incubate overnight at 37 °C at 230 RPM to facilitate production of S2-E inclusion bodies. Cultures were harvested the next morning by spinning cultures down at 3500 × g for 20 min at 4 °C and the pelleted cells were flash frozen in liquid nitrogen before being stored at –80 °C. Six liters of culture typically resulted in a wet cell mass of 10-14 g.

Purification of the SARS-CoV-2 Envelope protein

10-14 grams of frozen cells were thawed at room temperature and suspended in 140 mL of lysis buffer—75mM tris(hydroxymethyl)aminomethane (Tris) pH 7.8, 300 mL NaCl, and 0.2 mM ethylenediaminetetraacetic acid (EDTA)—supplemented with 0.5 mM magnesium acetate, lysozyme, ribonuclease, deoxyribonuclease, and 50 µL/gram wet cell mass protease inhibitor (P8849 Sigma). The lysis slurry was tumbled for two hours at 4 °C before being sonicated (Fisher Scientific, model FB705) on ice for 12 minutes at 60% power with alternating 5 second pulses and pauses (total power imparted on the slurry was ~ 80 kJ). The sonicated slurry was centrifuged at 25,000 × g for 20 minutes at 4 °C and the supernatant was discarded. The inclusion body pellets were resuspended with a Dounce homogenizer in 140 mL of lysis buffer. The sonication and centrifugation steps were repeated once in order to further clean the inclusion bodies. Clean inclusion body pellets were resuspended in 140 mL of lysis buffer supplemented with 3 % w/v N-dodecyl-N,N-dimethylglycine (Empigen), 0.5 mM dithiothreitol (DTT), and 25 µL/gram wet cell mass protease inhibitor before being tumbled overnight at 4 °C. The next morning, persistently insoluble materials were removed from the dissolved inclusion bodies by spinning at 25,000 × g for 45 minutes at 4 °C. The supernatant was saved.

2 mL of HisPur Ni-NTA Superflow agarose resin (Thermo Scientific) was equilibrated in lysis buffer before being mixed with the supernatant. The resin and supernatant were tumbled 1-2 hours at 4 °C before the resin was loaded onto a gravity column connected to an A_{280} detector. To wash away unbound impurities, 15 column volumes (CV) of 0.3% Empigen, 1×Tris-buffered saline (TBS, 20 mM Tris pH 7.5 140 mM NaCl), plus 0.25 mM DTT were passed through the resin. Low-affinity impurities were washed away with 15 CV washes of 30 mM imidazole, 1× TBS, 0.1% 1-myristoyl-2-hydroxy-sn-glycero-3-phosphocholine (LMPC), and 0.25 mM DTT. This was repeated for 75, 90, and 120 mM imidazole washes in order to remove the remaining impurities. A 10 CV solution of 0.2 wt% PMAL-C8 (Anatrace, Maumee, OH) in 1X TBS was washed over the resin in 2 CV pulses in order to remove the majority of the detergent and exchange the resin-bound envelope protein into amphipols. Excess amphipols and residual detergent were then removed by washing the column with 10 CV of 1× TBS. Amphipol-complexed envelope protein was then eluted from the column with 5 CV of 250 mM imidazole in 1× TBS pH 7.8. It was seen that envelope protein could be eluted in either amphipol PMAL-C8 or A8-35 (Anatrace), but PMAL-C8 was chosen because it is zwitterionic and easier to work with than the anionic A8-35. The S2-E construct tag can be proteolytically removed with thrombin in detergent solutions for structural and biochemical studies, but this was not carried out here because the thrombin precipitated the S2-E in PMAL-C8 amphipols solutions.

Fluorescent labeling of S2-E

Immediately after the envelope protein was eluted in complex with PMAL-C8, 1.5 mg of thiol-reactive N,N'-dimethyl-N-(iodoacetyl)-N'-(7-nitrobenz-2-oxa-1,3-diazol-4-yl)ethylenediamine (IA-NBD) (Thermo Fisher) or AFDye 488 Maleimide (Flouroprobes) was dissolved into 300 µL of dimethyl sulfoxide (DMSO) and added to the ~10 mL of eluted protein for labeling of one or more of its 3 cysteine residues (which are sequentially proximal within the S2-E extramembrane C-terminus). The reaction tube was covered in foil and tumbled at room temperature for one hour before being filtered through a 0.8 µm Acrodisc low-

protein binding filter (PALL PN 4618). The filtered amphipol/S2-E solution was then extensively dialyzed against 1× TBS pH 7.8 with 0.25 mM tris(2-carboxyethyl)phosphine hydrochloride (TCEP) and EDTA in 6-8 kD molecular weight cut-off (MWCO) dialysis tubing from Spectra/Por (part number 132660). On the morning of tissue culture treatment, the dialyzed protein was concentrated ~10× in a 10kD MWCO Amicon Ultra-15 filter cartridge by centrifuging at 2500 × g at room temperature (RT). Protein purity was checked by sodium dodecylsulfate polyacrylamide gel electrophoresis (SDS-PAGE) and the concentration was determined by measuring absorbance at 280 nm using an extinction coefficient of 6000 $M^{-1}cm^{-1}$. AFDye 488 Maleimide dye has absorbance at A280 and so the protein concetration was determined by reference on SDS-PAGE against unlabeled or NBD labeled E. Dye labeling efficiency was checked by measuring absorbance at 472 nm or 493 nm with an excitation coefficient of 23700 $M^{-1}cm^{-1}$ or 72000 $M^{-1}cm^{-1}$ for NBD or AF488 respectively. It was seen that the labeling efficiency was usually ~0.7 flourophore per protein, indicating that, on the average, only one of three wild type cysteine residues was modified. Dye-modified S2-E (S2-E-Dye) was normally used within one week (with storage at 4 °C) following completion of preparation. Indeed, we observed that preparations that were stored for longer than 3 weeks at 4 °C led to cellular results in which S2-E-NBD was seen to traffic as usual to the perinuclear space but did not then obviously segregate to one side of the nucleus at longer timepoints (16/24h), as was seen when freshly prepared samples were employed. Starting with six liters of culture, this protocol yields roughly 500 µg of fluorescently labeled S2-E protein.

Functional delivery of SARS-CoV-2 envelope protein from amphipols to planar bilayers

Planar lipid bilayers were formed from a solution of synthetic 1-palmitoyl-2-oleoyl-glycero-3-phosphocoline (POPC) and 1-palmitoyl-2-oleoyl-glycero-3-phosphoethanolamine (POPE, Avanti Polar Lipids) in a 3:1 mole ratio in *n*-decane (Sigma-Aldrich). A glass bulb was used to apply the POPC/POPE solution in a 150 µm aperture of a Delrin cup (Warner Instruments), separating the *cis* and *trans* chambers. Asymmetric buffers were used, with 5 mM 4-(2-hydroxyethyl)-1-piperazineethanesulfonic acid (HEPES), 500 mM NaCl, pH 7.2 for the *cis* chamber, and 5 mM HEPES, 50 mM NaCl, pH 7.2 for the *trans* chamber as done in previous studies for comparison.(1,2) The bilayer capacitance ranged between 50-65 pF.

Delivery of the SARS-CoV-2 envelope protein (S2-E) complexed by PMAL-C8 amphipols to pre-formed planar bilayers was achieved spontaneously by pipette addition of ~1 µL of ~3 µM of the S2-E–amphipol complex and accompanied by stirring of the chambers. Each chamber (cis/trans) had 2.5 mL of buffer (5mL total volume). Incorporation of S2-E was achieved by addition of PMAL-C8 complexed S2-E to either the cis or to both cis and trans chambers. The final concentration of protein varied from ~5 nM–15 nM. S2-E unitary currents were collected from a BC-535 amplifier (Warner Instruments) at 23 ± 1°C, 10 mV/pA gain and low-pass filtered by a 4-pole Bessel filter at 1 kHz. The resulting data were digitized using an analog-to-digital converter (Digidata 1550A, Molecular Devices) at 1 kHz using the pClamp10.7 software suite (Molecular Devices). The single-channel data were collected over separate amphipol delivery experiments carried out on different days, with three-minute recordings for each voltage. Recordings were analyzed using the single-channel search in ClampFit 10.7 (Molecular Devices) filtered with a 100 Hz low pass Gaussian filter and ignoring short level changes of 25 ms duration to obtain the number of events, open probability, reversal potential, and single-channel conductance. Similar conditions were conducted with the S2-E-NBD. Control experiments where PMAL-C8 only (no S2-E) was added to the planar bilayers did not exhibit channel activity at 1x or 50x amphipol concentrations relative to those used in S2-E and S2-E-NBD planar bilayer experiments.

Maintenance of cell cultures

Human cell lines HeLa (ATCC cat# CCL-2) and alveolar SW1573 (ATCC cat# CRL-2170) cells were obtained from ATCC. HeLa cells were maintained in a tissue culture incubator with humidified air supplemented with 5% CO_2 at 37 °C. HeLa cells were grown in low glucose Dulbecco's Modified Eagles Medium (DMEM, Gibco cat# 11885-084) supplemented with 10% fetal bovine serum (FBS, Gibco 26140-079), 1% penicillin/streptomycin (P/S, Corning, Ref 30-0020CI). SW1573 cells were kept in a tissue culture incubator with humidified air without added CO_2 at 37°C. The SW1573 were grown and maintained in (Leibovitz's medium # 15, or L-15; ATCC cat # 30-2008) supplemented with 10% FBS and 1% P/S. Both cell lines were passaged 2 times a week and seeded such as to always remain below 95% confluency.

Cell culture treatments

The underlying idea of our S2-E-to-cell experiments was to treat cells with aliquots of fluorescently-tagged S2-E/amphipol and then to follow cell association and intracellular trafficking of the flurorescently-taged protein over time. These experiments were conducted with healthy growing cells that are well attached to a glass coverslip surface. These cells were treated with various concentrations of SE-2 protein/amphipol for differing amounts of time. This is achieved with freshly concentrated, and preferably freshly made, SE-2 protein complexed with amphipol PMAL-C8 in TBS. In order not to perturb the cells by changes in osmotic pressure, pH, or temperature the stock S2-E amphipol solution was processed prior to addition to the cells. S2-E was first dialyzed against TBS. Further, the resulting S2-E/amphipol solution was added to fresh 37 °C pre-warmed cell media and mixed before aliquots were then added to cells. The added volume of the S2-E/amphipol solution added to the cell culture never exceeded 10% of the culture volume and was often less (4-6%). At the chosen time points, the coverslip containing attached cells was rinsed to discard floating cell debris and then 'fixed' with paraformaldehyde. Fixing is intended to chemically freeze cells. The procedure kills the cells but preserves their plasma membrane surface and intracellular structures. After discarding excess fixative, cells are treated differently depending on the cell structures we wished to observe in relation to S2-E protein. When the goal was to see S2-E in relation to the plasma membrane we used WGA a lecithin conjugated to a fluorophore together with a cell nucleus dye DRAQ5 which preferentially binds double-stranded DNA. When the intent was to observe intracellular organelles Golgi,ERGIC, and others the fixed cells were permeabilized by incubation with detergent with the goal being to make small holes of sufficient size, for antibodies specific to those structures, to enter the cell surface and gain access to Golgi,ERGIC, and other cell organelles.

Exponentially growing cells were plated in the wells of a 12-well plate with 1.5 thickness glass coverslips (Fisherbrand cat # 12-545-81) at a cell density of 20-23,000 live cells/well in 2mL cell media. After a day, the cells destined for 24h and 16-18h time points were treated by rinsing once with Dulbecco's phosphate buffered saline without calcium and magnesium (DPBS, Corning cat 21-031-CV) and by adding 1 mL of fresh medium with no more than 5-10 % volume of freshly concentrated S2-E protein (labelled or not) with amphipol in the dialysis buffer at the concentrations stated in the figure captions. Cells were protected from light. The remaining and undiluted S2-E/amphipol stock was stored at 4 °C overnight protected from light and used in the same way the next morning for the remaining time points. At the appropriate time, each coverslip was transferred into a fresh 12-well plate well facing upwards containing 2 mL of 37 °C pre-warmed DPBS. DPBS was discarded by suction, and cell fixation was started by adding gently 1 mL/well of pre-warm 4% paraformaldehyde (PFA, EMS cat #15714) dissolved in DPBS for 15 min at 37 °C. After fixation in 4% PFA, all steps were carried out with gentle mixing and at room temperature and with plates protected from light by being kept wrap in aluminum

foil. All solutions were filtered prior to addition to coverslips to avoid addition or formation of aggregates. Fixation was stopped by discarding the 4% PFA solution by suction and by gently rinsing 2 times with 2 mL/well with DPBS for 15 min/each. The cell membrane was labeled with wheat germ agglutinin conjugated to Alexa Fluor-555 (WGA-AF555, Invitrogen cat# W32464) at a final concentration of 5 µg/mL for 15 min using 1 mL/well. Coverslips were then rinsed from excess WGA-AF555 by rinsing 2 times with 2 mL/well in DPBS for 15 min for each time point and once using 100mM glycine in DPBS. The times of cell treatment were imperfectly staggered. After the 100 mM glycine rinse, the timepoint samples were set aside without shaking until other time points were completed. When all time points had finished the 100 mM glycine rinse, all coverslips were transferred into a common 12-well plate, and the following treatments proceeded as a group.

For time-course S2-E-NBD trafficking course experiments without antibodies (Figs. 2 and S6) cells were cell nucleus-labeled using freshly opened of DRAQ5 (Invitrogen cat #65-0880), an anthraquinone membrane permeable dye with high affinity for double-stranded DNA. DRAQ5 was used diluted 1:2500 in DPBS using 2 mL/well and incubated for 1 h. Excess DRAQ5 was then rinsed away twice with 2mL/well 5-10 min each DPBS +0.01% Triton X-100 (TX-100) and once with DPBS only. Coverslips were mounted in glass slides using Prolong Gold antifade (Invitrogen, cat # P10144) and cured, typically for 2 days, before imaging.

For experiments requiring antibody detection of organelle-specific marker proteins (Figs. 3, S10-S12, and S14-S16) or anti-E (Figs. S5 and S7), cells were permeabilized with 1 mL/well containing 0.1% TX-100 dissolved in DPBS for 15 min. Permeabilization was stopped by rinsing twice in 2 mL/well DPBS and blocking overnight with 2 mL/well of 1% BSA dissolved in DPBS with 0.01% TX-100. The day after, plates were allowed to reach RT for 30-40 min without mixing and then treated with specific antibodies for detection of Golgin97 (1 mL/well, diluted 1: 500; Invitrogen, cat #A21270), or ERGIC53 (3) (1 mL/well, diluted 1:500; ENZO cat# ENZ-ABS300), or for E protein detection (1:1000 dilution of rabbit polyclonal antibody ProSci cat# 10-518, (this polyclonal was not very specific and weakly reacts to a nuclear protein in HeLa cells, see Fig. S5 panel E) or KDEL (1mL/well diluted 1:400; Abcam, cat# EPR12668) or GM130 (1mL/well diluted 1:500; Cell Signaling, cat #12480S) or EEA1 (1mL/well diluted 1:450; Invitrogen, cat # MA5-14794) or Lamp1 (1mL/well diluted 1:400; Encorbio, cat # MCA-5H6) or vimentin (1mL/well diluted 1:500; Cell Signaling, cat #5741S. All antibodies were diluted in freshly made and 0.2 µm filtered 1% BSA dissolved in DPBS + 0.01% TX100 (1%BSA)and incubated for 2 h at RT with gentle mixing. Each coverslip was treated to remove unbound primary antibody by rinsing 3 times with 2mL/well DPBS + 0.01% TX-100, 15 min each. Samples were then incubated with a secondary antibody conjugated to a fluorescent dye using 1 mL/well for 1 hr in 1% bovine serum albumin (BSA) using 1:1000 dilution of goat anti-mouse conjugated to Alexa Fluor 546 (AF546; Invitrogen cat# A11030), against mouse mAb for Golgin-97 and ERGIC53. For samples treated with rabbit anti-E polyclonal (in Fig. S7) we used donkey anti-rabbit conjugated Alexa Fluor 488 (AF488; Invitrogen cat# A32790). For Figures S5, S10-S12, S14 and S16 where samples were treated with rabbit antibodies and we used donkey anti-rabbit conjugated Alexa Fluor 555 (Invitrogen cat # A31572),while for anti-Lamp1 (Fig. S15) we used goat anti-mouse conjugated Alexa Fluor 555 (Invitrogen cat # A32727). After 1 h incubation with gentle shaking the samples were rinsed twice for 15 min with 2 mL/well DPBS + 0.01% TX100 and treated in the same way as above for nuclear counter-staining using DRAQ5 and coverslip mounting.

For live-cell imaging experiments, HeLa cells exponentially growing and maintained as above were split into a 12-well plate (Corning # 3512) or 35 mm glass bottom petri dishes (MatTek Part No. P35G-1.5-12-C) at a density of 5000 cells/cm^2 the day before treatment and grown overnight in 5% CO_2 atmosphere. The next day, cells were rinsed once with RT DPBS and their cell culture media was exchanged for 1 mL

pre-warmed L15 complete medium and placed in a 0% CO_2 incubator for 30 minutes. After thermal equilibration to L15 media, samples imaged within 3 hours were treated with 10 µg /mL of WGA-555, and for nuclear labeling used 1-drop/mL Hoechst 33342 (Invitrogen, cat # R37605), and 2.5 µM of either AF488 or NBD labeled S2-E in PMAL-C8 was added before being placed back in the 0% CO2 incubator for the first hour For samples imaged 24 h after after treatment with S2-E-label (data not shown), WGA-AF555, and the nuclear stain were added one hour before imaging. Samples were imaged both before and after washing away labeling media (S2-E, WGA-555, and NucBlue) in order to ensure that free S2-E did not interfere with imaging (see Fig. S4).

For live cell imaging with cysteine-NBD (Fig. S8), HeLa cells growing in low glucose DMEM were split into 35 mm glass bottom petri dishes (MatTek Part No. P35G-1.5-12-C) at a density of 5000 cells/cm^2 and grown for ~36 hours in 5% CO_2. The cysteine-NBD was created the morning before the HeLa cell treatment. One mg of IA-NBD amine was reacted with two mg of L-cysteine amino acid in one mL of 80/20 water/DMSO and reacted in the dark at RT for one hour. The resulting mixture was filtered with a 0.8 µm Acrodisc low-protein binding filter and the NBD concentration was determined by UV-Vis as with S2-E. Cells were treated with 5 µM cysteine-NBD and placed back in the incubator before imaging. Prior to imaging, media was replaced with 1 mL of dye-free pre-warmed complete DMEM low glucose media.

Cell imaging

All confocal experiments shown were imaged using a LSM 510 confocal microscope, with a Plan-Neofluar 40x/1.30 Oil DIC objective. The confocal pinholes were set at 84µm for the 633 nm HeNe1 laser line, at 80µm, for the 543 nm HeNe1 laser line, and at 86µm for the Argon laser. These settings correspond to a pinhole diameter of 1.00 Airy units for the HeNe2 laser (633 nm), and the same optical slice of 1.1 µm for all three lines. These settings were kept for all channels in all experiments. The fluorophores were excited using the 488 nm line of a 40 mW Argon laser set at 10% power (reduced at source to 50%, for a final 5%) for NBD and AF488 signal. The 543 nm line was set at 10% power for WGA-AF555 and for anti-mouse AF546, whereas the 633 nm line of a HeNe laser was set to 15-50% power for DRAQ5 depending on the dye emission intensity. Images were collected at 1-2× and occasionally 3× zoom. The frame size was set usually to 1024, data depth was 8 bit, and Multi track/frame mode was used, for each channel. The scan average parameter was usually set to 4, but sometimes to 8 or 16. For presentation purposes, images were processed using ImageJ software.

Live cell imaging was done with an ECHO Revolve equipped with a ELWD Universal Condenser; NA 0.30 WD 73m and using a 40x PLAN Fluorite LWD CC Phase Ph2 NA 0.60 objective. LED light cubes used were: DAPI - EX:385/30 EM:450/50 DM:425, for the nuclear stain; FITC - EX:470/40 EM:525/50 DM:495 for the E-labeled protein, and TxRED - EX:560/40 EM:635/60 DM:600 for WGA-AF555.

Statistics

The numbers of replicates, validation of reagents, software, and statistical approaches are detailed in the above methods and/or in the relevant figure captions.

Supporting References

1. Wilson, L., McKinlay, C., Gage, P., and Ewart, G. (2004) SARS coronavirus E protein forms cation-selective ion channels. *Virology* **330**, 322-331
2. B. Xia, Y. He, X. Pan, F.-L. Liu, Y. Wang, S. Fang, Y. Wu, Z. Duan, X. Jiang, L. Xu, H. Chi, S. Li, Q. Meng, H. Zhou, Y. Zhou, X. Cheng, X. L. Xin, H.-L. Zhang, D.-D. Yu, M.-H. Li, X. L. Feng, J. Chen, H. Jiang, G. Xiao, Y.-T. Zheng, L.-K. Zhang, J. Shen, J. Li. and Z. Gao, SARS-CoV-2 envelope protein causes acute respiratory distress syndrome (ARDS)-like pathological damages and constitutes an antiviral target., Cell Res., 2021, doi:10.1038/s41422-021-00519-4.
3. Venkatagopalan, P., Daskalova, S. M., Lopez, L. A., Dolezal, K. A., and Hogue, B. G. (2015) Coronavirus envelope (E) protein remains at the site of assembly. *Virology* **478**, 75-85
4. Feryforgues, S., Fayet, J. P., and Lopez, A. (1993) Drastic Changes in the Fluorescence Properties of Nbd Probes with the Polarity of the Medium - Involvement of a Tict State. *J Photoch Photobio A* **70**, 229-243
5. Rezgui, R., Blumer, K., Yeoh-Tan, G., Trexler, A. J., and Magzoub, M. (2016) Precise quantification of cellular uptake of cell-penetrating peptides using fluorescence-activated cell sorting and fluorescence correlation spectroscopy. *Biochim Biophys Acta* **1858**, 1499-1506
6. Pavelka, M., Neumuller, J., and Ellinger, A. (2008) Retrograde traffic in the biosynthetic-secretory route. *Histochem Cell Biol* **129**, 277-288
7. Kanazawa, T., Takematsu, H., Yamamoto, A., Yamamoto, H., and Kozutsumi, Y. (2008) Wheat germ agglutinin stains dispersed post-golgi vesicles after treatment with the cytokinesis inhibitor psychosine. *J Cell Physiol* **215**, 517-525

Supporting Figures

Figure S1

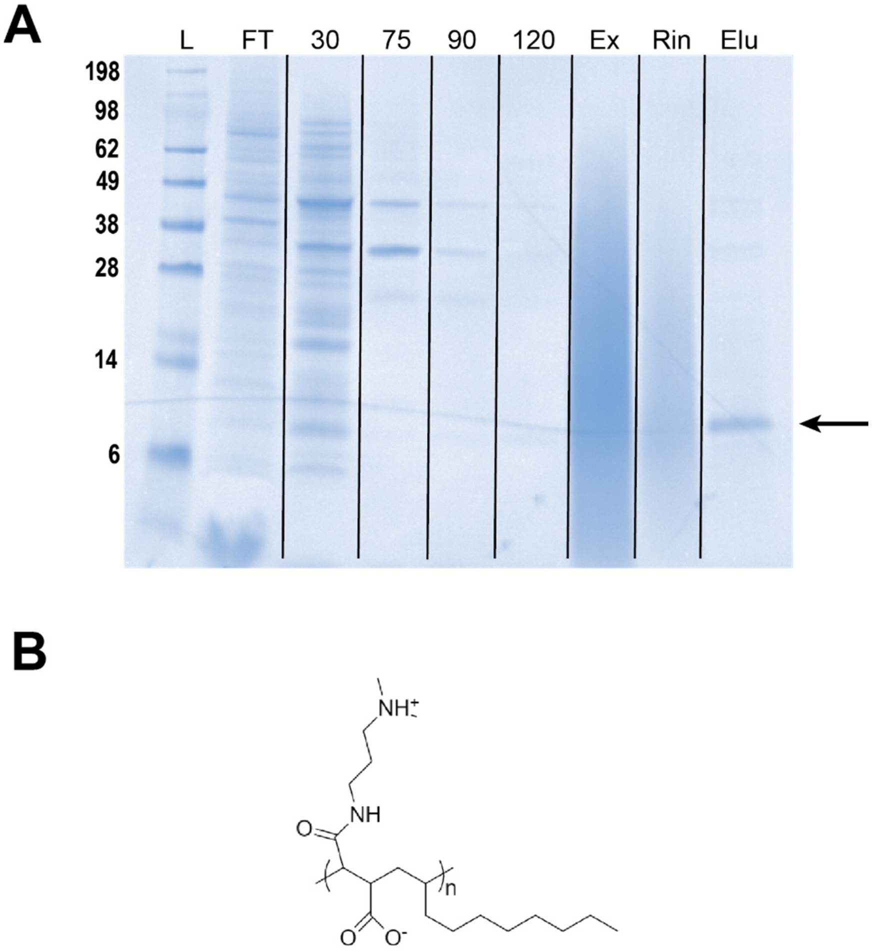

Figure S1. Representative SDS-PAGE of SARS-CoV-2 E (S2-E) purified into PMAL-C8. A) Each lane represents a step in the purification protocol after passing detergent-solubilized inclusion bodies over the column. SDS-PAGE samples were boiled and reduced with 5mM DTT before being run. Gels were stained with the SimplyBlue coomassie alternative (Invitrogen). Lanes from left to right are: Molecular Weight Standard **L**adder SeeBlue Plus2, **F**low-**T**hrough, **30** mM imidazole wash, **75** mM imidazole wash, **90** mM imidazole wash, **120** mM imidazole wash, **Ex**change into PMAL-C8, **Rin**se unbound PMAL-C8, and **Elu**tion. The black arrow points to the purified S2-E protein in the final elution. **B)** Chemical structure of PMAL-C8.

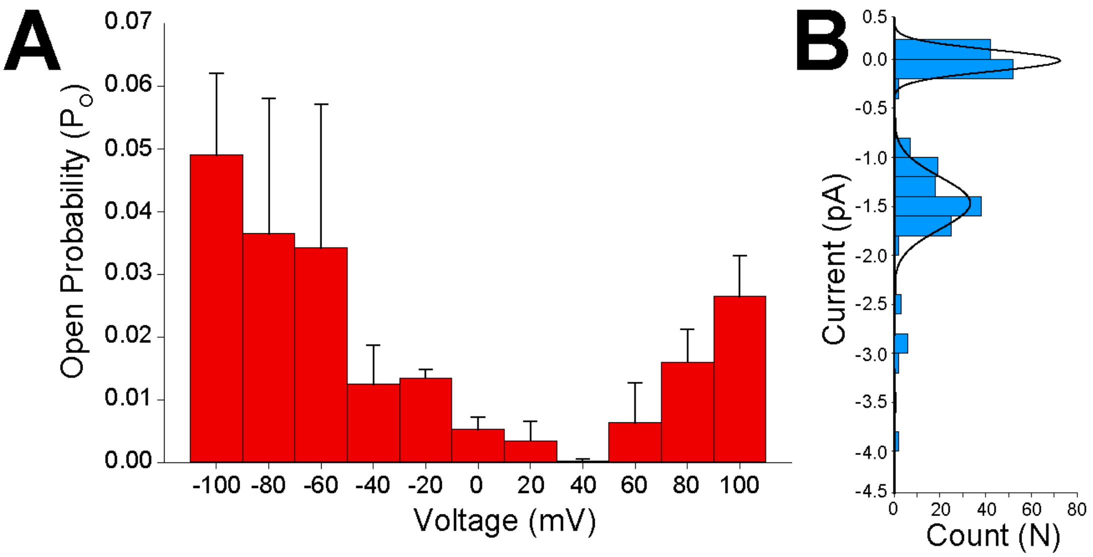

Figure S2. S2-E ion channel open probability and current amplitude distribution from planar bilayer electrophysiology measurements after amphipol delivery. Previous studies of CoV E proteins indicate that the channel has low open probabilities and current amplitudes. **(A)** Shows the open probability as a function of voltage for the data in Fig. 1*C*. Error bars are SEM from three distinct delivery experiments on different days and in total correspond to 9 min of measurement per voltage. **(B)** Identifies the number of events and the respective currents recorded at -100 mV. The histogram was fit to a 3 polynomial Gaussian fit with an average amplitude of 1.47 ± 0.05 pA for the open state. As in panel A, these data are from three separate recordings.

Figure S3

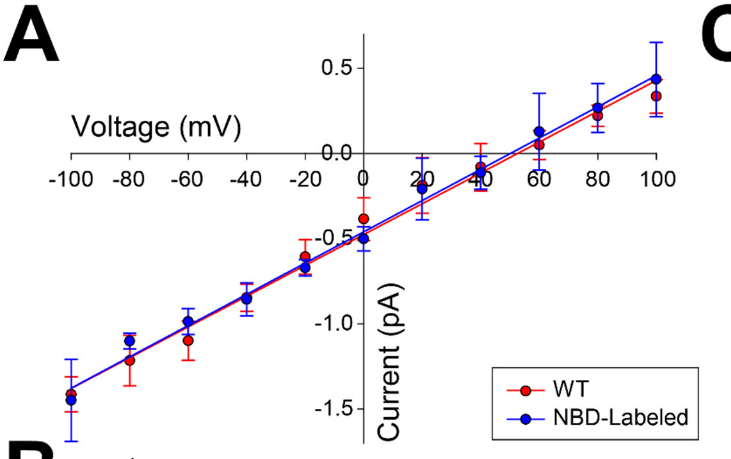

B

	Conductance (pS)	Reversal Potential (mV)
WT	9.0 ± 0.3	53 ± 3
NBD-Labeled	9.2 ± 0.2	50 ± 2

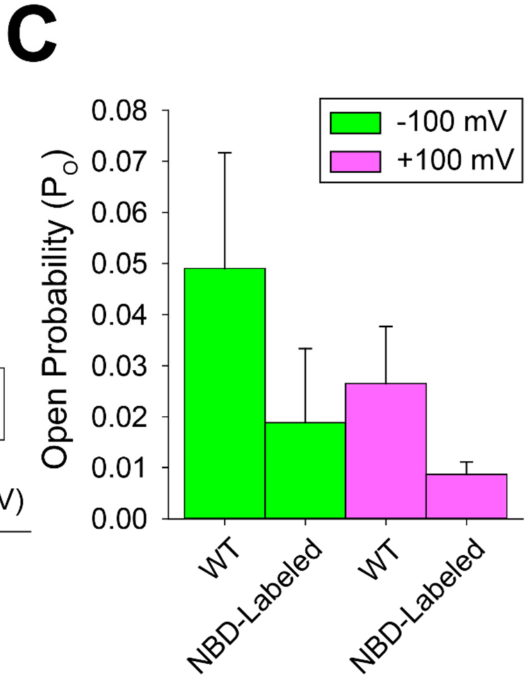

Figure S3. Planar lipid bilayer electrophysiology characterization of NBD-labeled SARS-CoV-2 envelope protein delivered from amphipols. (A) Comparison of unlabeled (WT) and NBD-labeled S2-E current-voltage relationship shows that NBD-labeling has no meaningful impact on conductance and reversal potential (B). (C) NBD-labeling of S2-E results in a nonstatistically significant decrease in open probability relative to the WT based on calculations of an unpaired t-test with P-values of 0.125 at -100 mV and 0.055 at 100 mV. Data represent three replicates. Error bars are standard deviations from three distinct delivery experiments on different days and correspond to a total measurement time of 9 min per voltage.

Figure S4

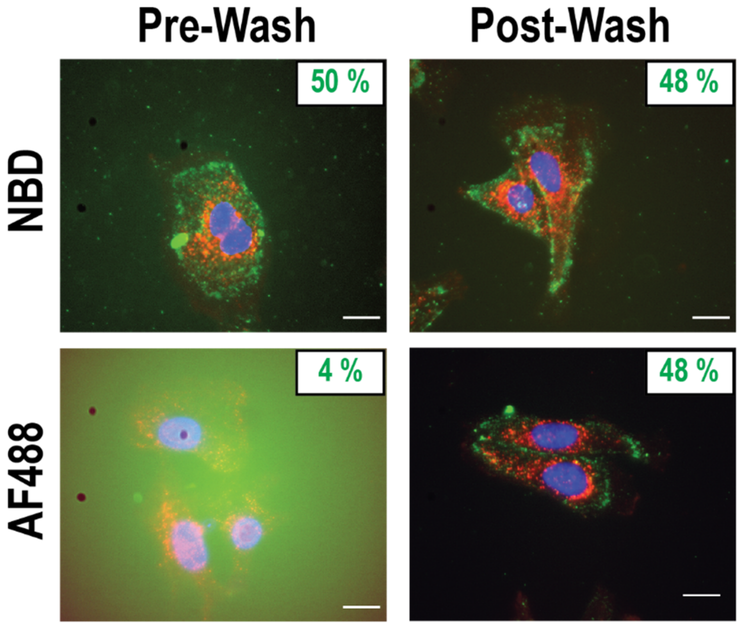

Figure S4. Comparison of live cell imaging when S2-E is labeled with either NBD or AF488. Top Left: The solvatochromic properties of NBD allows for live cell imaging of amphipol-mediated S2-E-NBD delivery to the plasma membrane. The S2-E-NBD has a low fluorescent intensity in bulk solution and this facilitates live cell imaging with the S2-E-NBD containing media--supplemental reference (4). While this is an undesirable characteristic for soluble targets it is suited for use with membrane proteins where the intensity of NBD is orders of magnitude higher when associated with the lipid bilayer. **Bottom Left:** AF488 is not solvatochromic and thus has a constant high fluorescent intensity, supplemental reference (5). The high background from S2-E-AF488 prevents live cell imaging of S2-E-AF488 delivery to the plasma membrane. **Top Right:** Washing away the S2-E-NBD containing medium and replacing it with fresh S2-E-NBD-free media does not significantly impact visualization of S2-E-NBD delivery to cells. **Bottom Right:** Washing away the S2-E-AF488 containing media and replacing it with fresh S2-E-AF488 free media drastically reduces background and allows adequate visualization of S2-E-AF488 delivery to cells. DAPI and TxRED settings were 110 ms at 16 % power and 620 ms at 81 % power respectively for all images. FITC settings were 570 ms, with power levels are indicated on each panel in the upper right hand corner. This experiment was performed twice with identical overall results. Scale bars are 25µm. Since S2-E is a membrane protein and given the ease of use, lower molecular weight, and lower cost of NBD; we preferencially used this label for most experiments. NBD use in membrane proteins appears validated by the comparable Pearson correlation coefficients results obtained using either S2-E-NBD and S2-E-AF488 (see captions for Figs. S5 and S10).

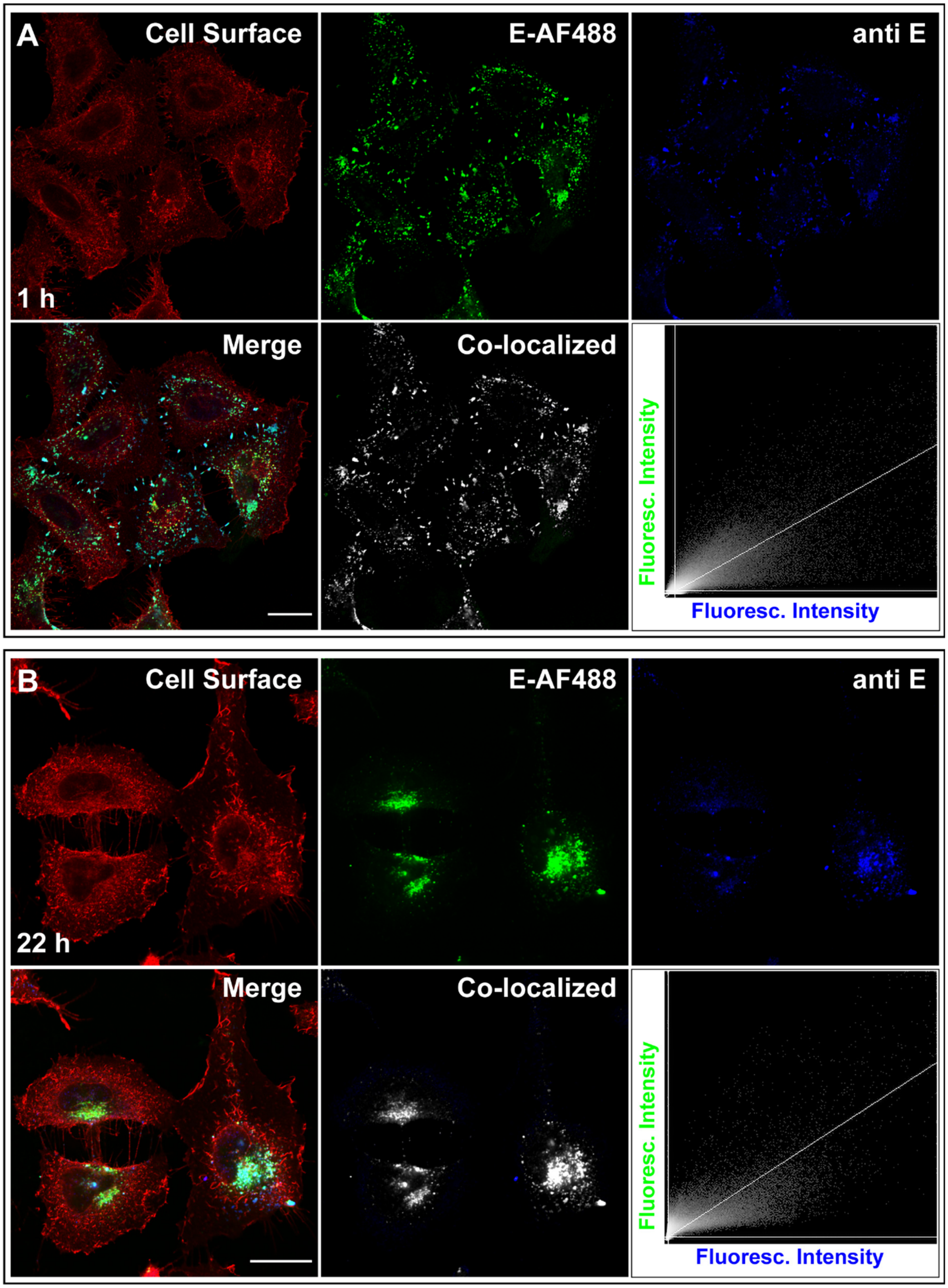
A
Cell Surface
E-AF488
anti E
1 h
Merge
Co-localized
Fluoresc. Intensity
Fluoresc. Intensity
B
Cell Surface
E-AF488
anti E
22 h
Merge
Co-localized
Fluoresc. Intensity
Fluoresc. Intensity

Figure S5. panels C and D (Second Page of Three-Page Figure)

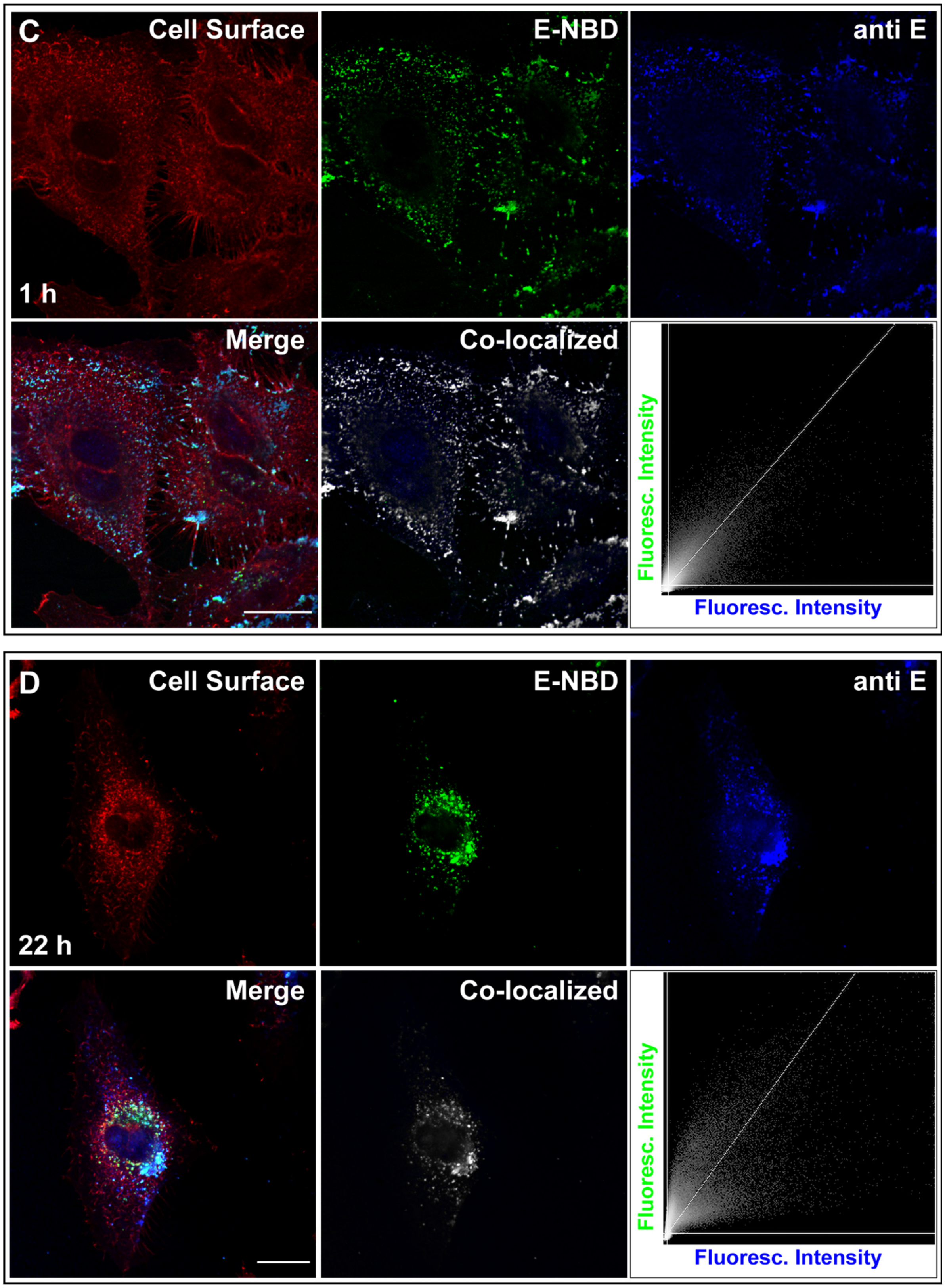

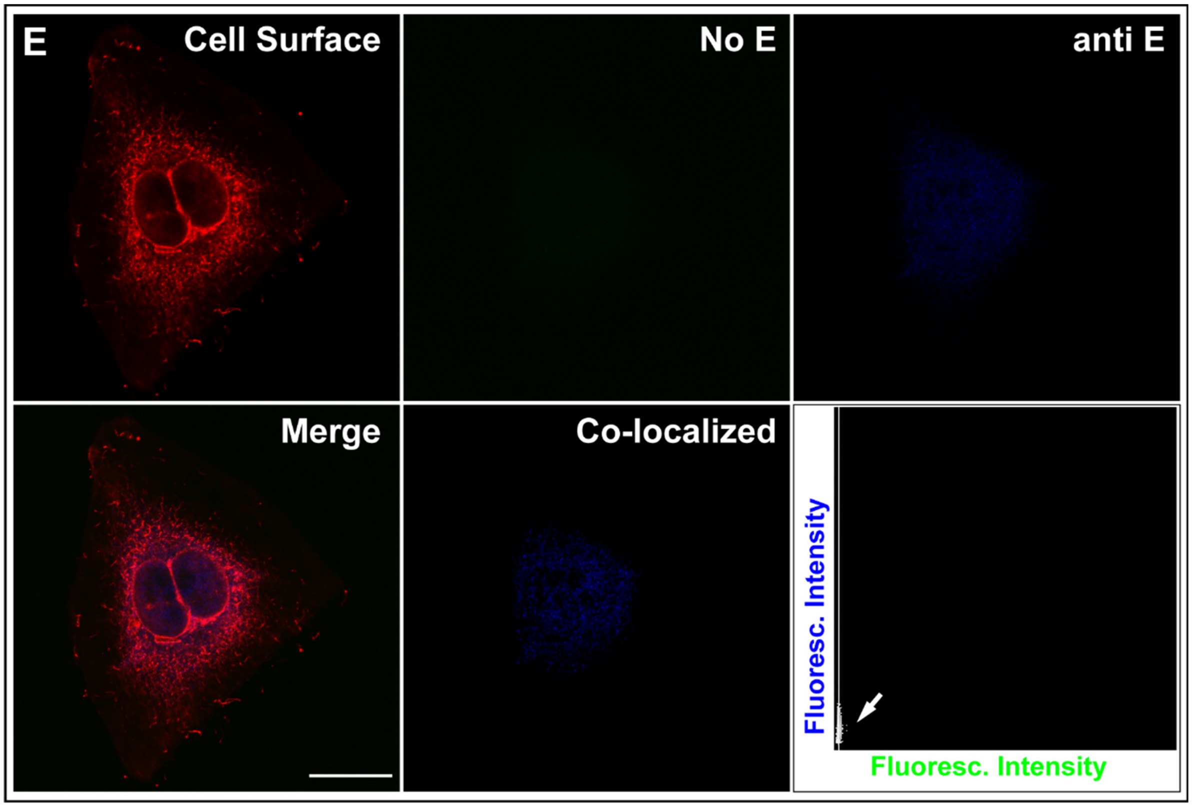

Fig. S5. (This page and 2 preceding pages). SARS-CoV-2 envelope protein labeling in cysteines with NBD or AF488 does not affect E retrograde trafficking. To validate the integrity of the fluorescent signals from S2-E-NBD or S2-E-AF488 over time, HeLa cell samples were treated with each labeled protein for 1 h or 24 h. After the indicated times,samples were fixed, membrane stained with WGA-AF555, and probed with polyclonal anti-E antibodies (ProSci cat#10-518; dilution1:1000). Panel **A** shows HeLa cells treated with 2.5µM S2-E-AF488 after 1 h. First row, individual color channels used to detect cell surface (red), S2-E-AF488 (green) and anti-E (blue). Second row shows the merged image of the above three channels, followed by the co-localized image in white between S2-E-AF488 (green) and anti-E (blue) images. The last image is the intensity plot between green and blue images. Colocalization image and intensity plots were obtained using the Image-J plugin Colocalization Threshold, without ROIs. Panel **B** shows cells treated with S2-E-AF488 for 24 h. Both rows in B are presented as in panel A. Panel **C** shows HeLa cells treated with 2.5µM E-NBD after 1 h. First row in C shows individual color channels used to detect cell surface (red), E-NBD (green) and anti-E (blue). Second row in C shows the merged image, followed by the co-localized image between S2-E-AF488 and anti-E, and the intensity plot between green and blue images. Panel **D** shows cells treated with S2-E-AF488 after 24 h. Both rows in D are presented as in panel C. Experiments shown in panels A-D were performed 3 independent times, twice using E-NBD and once with S2-E-AF488. For each E-labeled time point, at least 5 images were recorded and analyzed for colocalization, using four Image-J plugins (Colocalization Test, Colocalization Threshold, Coloc-2, and JACoP). As expected, all colocalized images in panels A-D show strong Pearson correlation with comparable coefficients. Using the colocalization-threshold plugin, average Pearson correlation coefficients with standard deviations for each experiment were: S2-E-NBD#1; 1 h, 0.72 ± 0.05 and 24 h, 0.68 ± 0.03; for S2-E-AF488; 1 h, 0.73 ± 0.09 and 24 h, 0.68 ± 0.05 and for S2-E-NBD#2; 1 h, 0.77 ± 0.05, 24 h, 0.70 ± 0.11. Panel **E** is HeLa cells non-

treated with S2-E but probed with anti-E antibodies showing that the antibodies used exhibit non-specific binding to a nuclear target. Note that the anti-E and co-localized images are enhanced to show the unspecific binding of the anti-E, and that HeLa cells treated with S2-E-NBD or S2-E-AF488 do not show green fluorescence in the cell nuclei. Scale bars are 20 µm. For more details see online Materials and Methods.

Figure S6

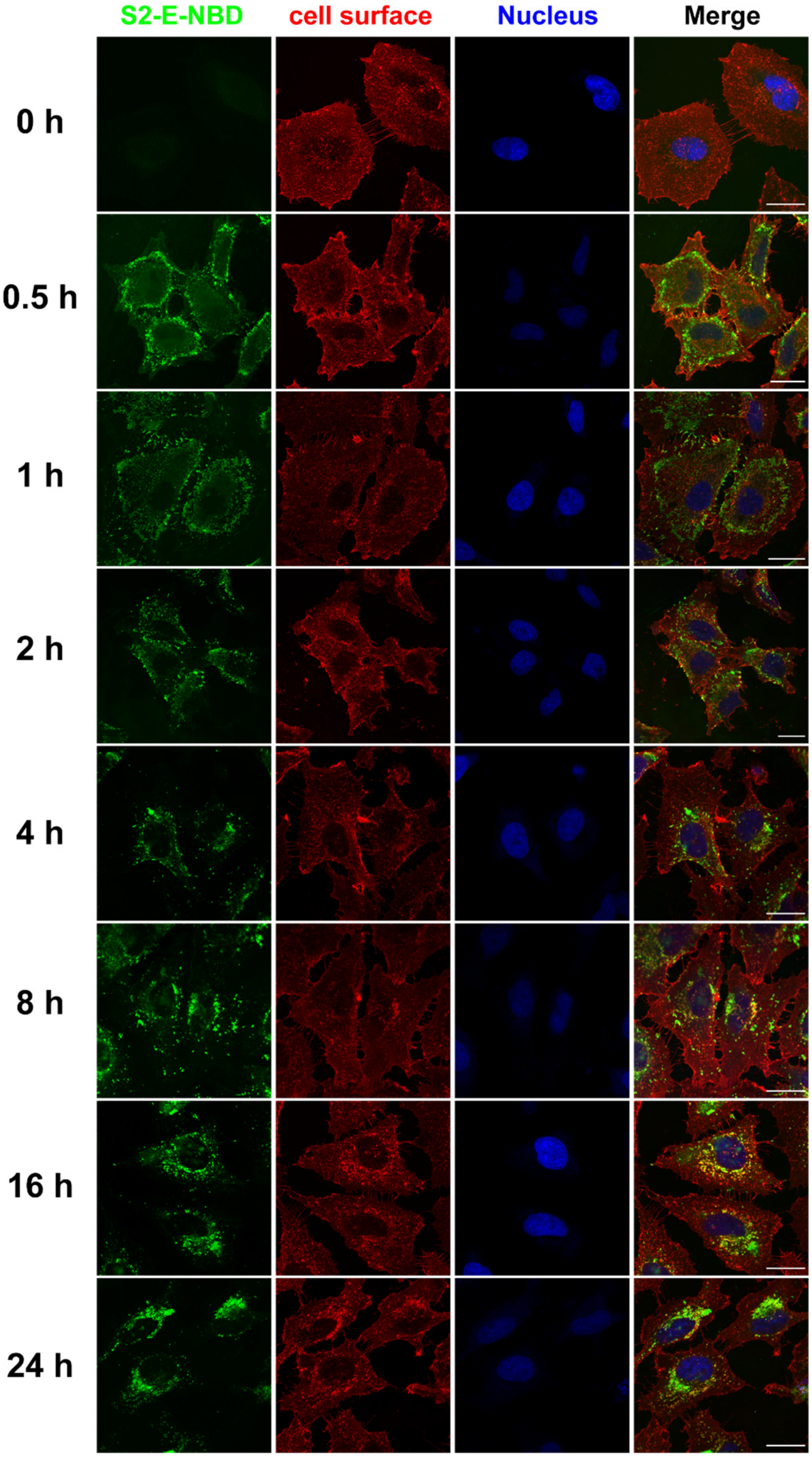

Figure S6. Individual channels for each composite image shown in Fig. 2 of the main text. Color scheme: green, NBD-labeled S2-E; red, cell surface plasma membrane (WGA-AF555); and blue, cell nuclei (DRAQ5). Note the movement over time of the S2-E-NBD signal towards one side of the cell nucleus while the red signal remains constant. Scale bar 25 µm.

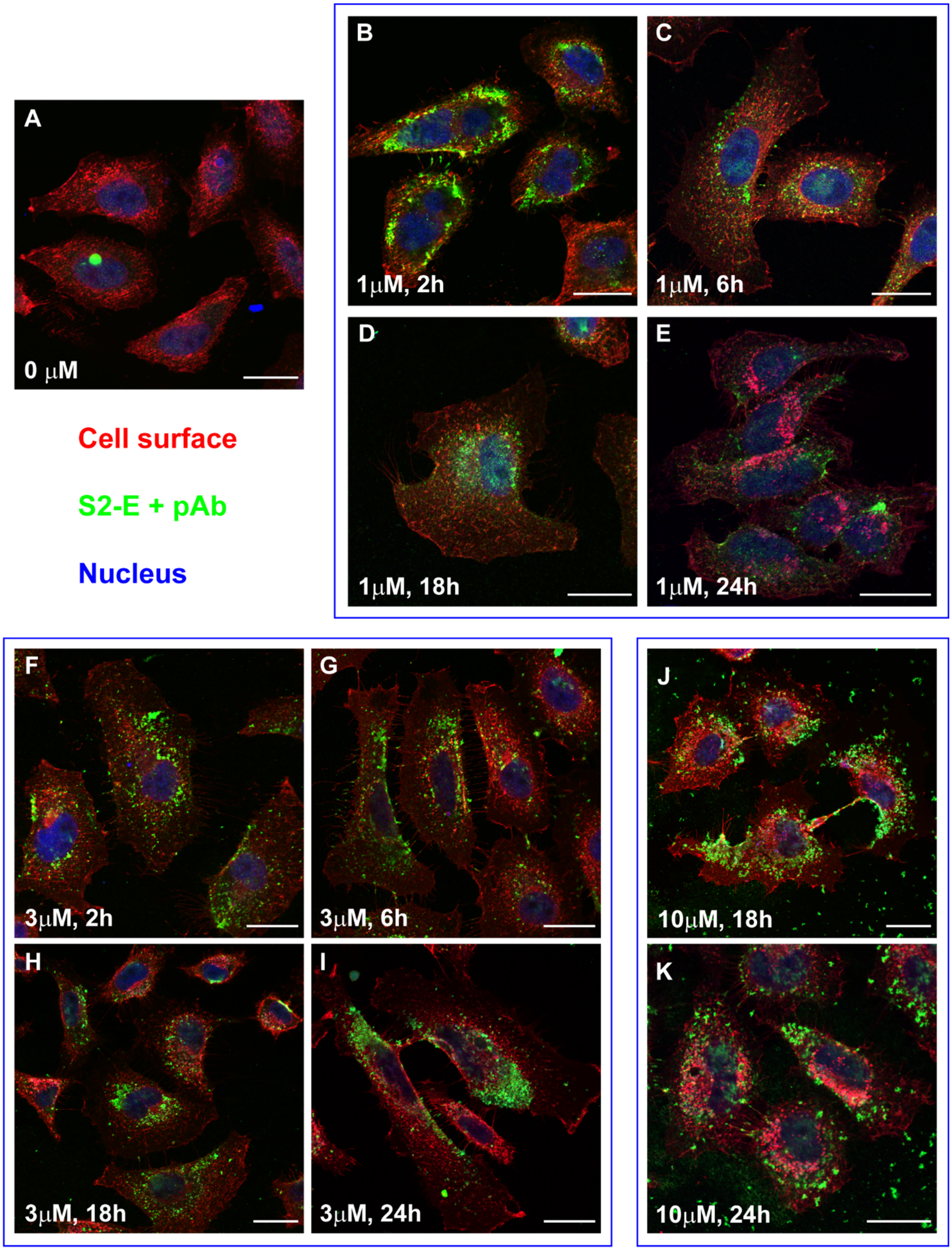
A
0 μM
Cell surface
S2-E + pAb
Nucleus
B
1μM, 2h
C
1μM, 6h
D
1μM, 18h
E
1μM, 24h
F
3μM, 2h
G
3μM, 6h
H
3μM, 18h
I
3μM, 24h
J
10μM, 18h
K
10μM, 24h

Figure S7. Lower Panel I. Individual channels and composite of upper panels A and B-E

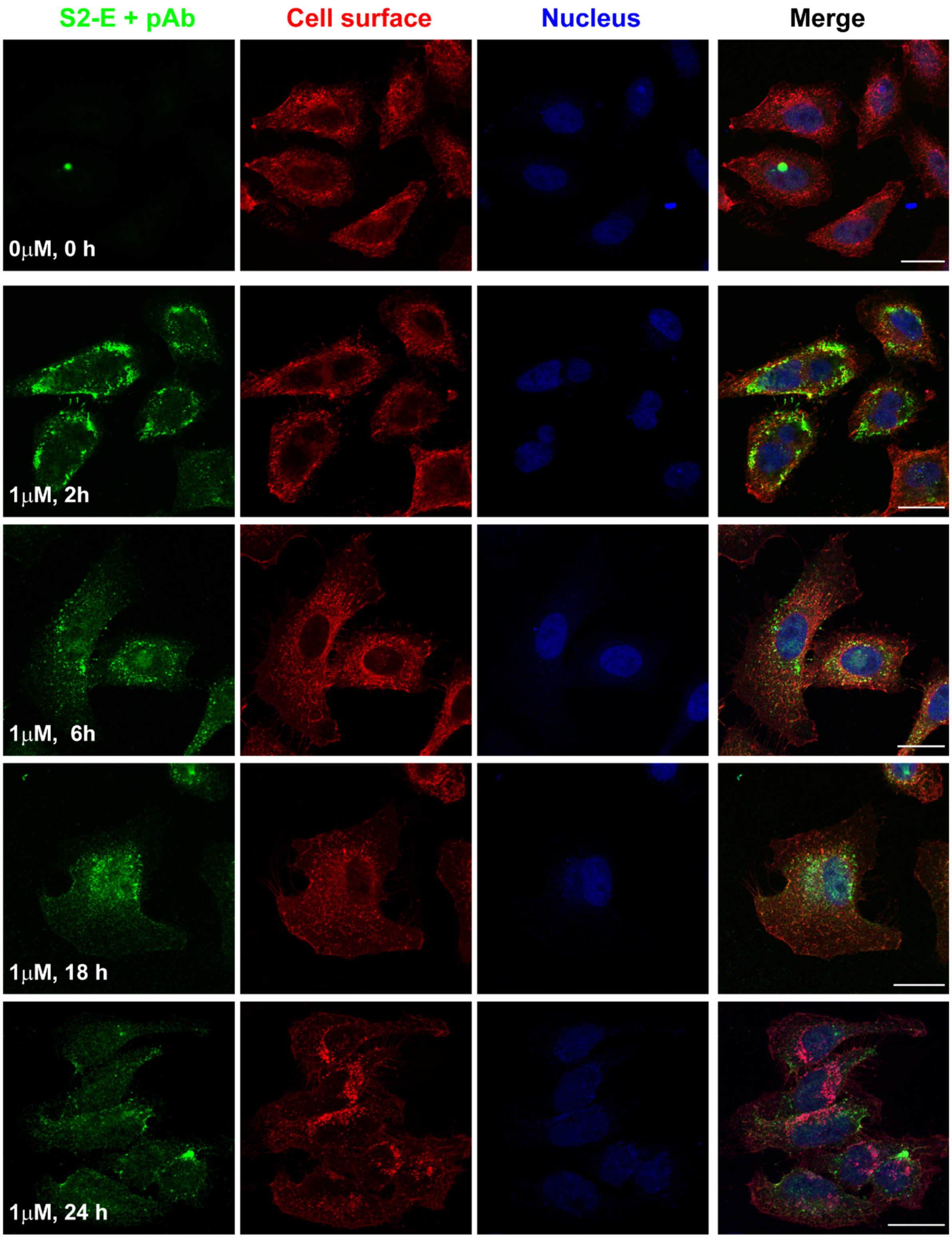

Figure S7. Lower Panel II. Individual channels and composite of upper panels A, F-I

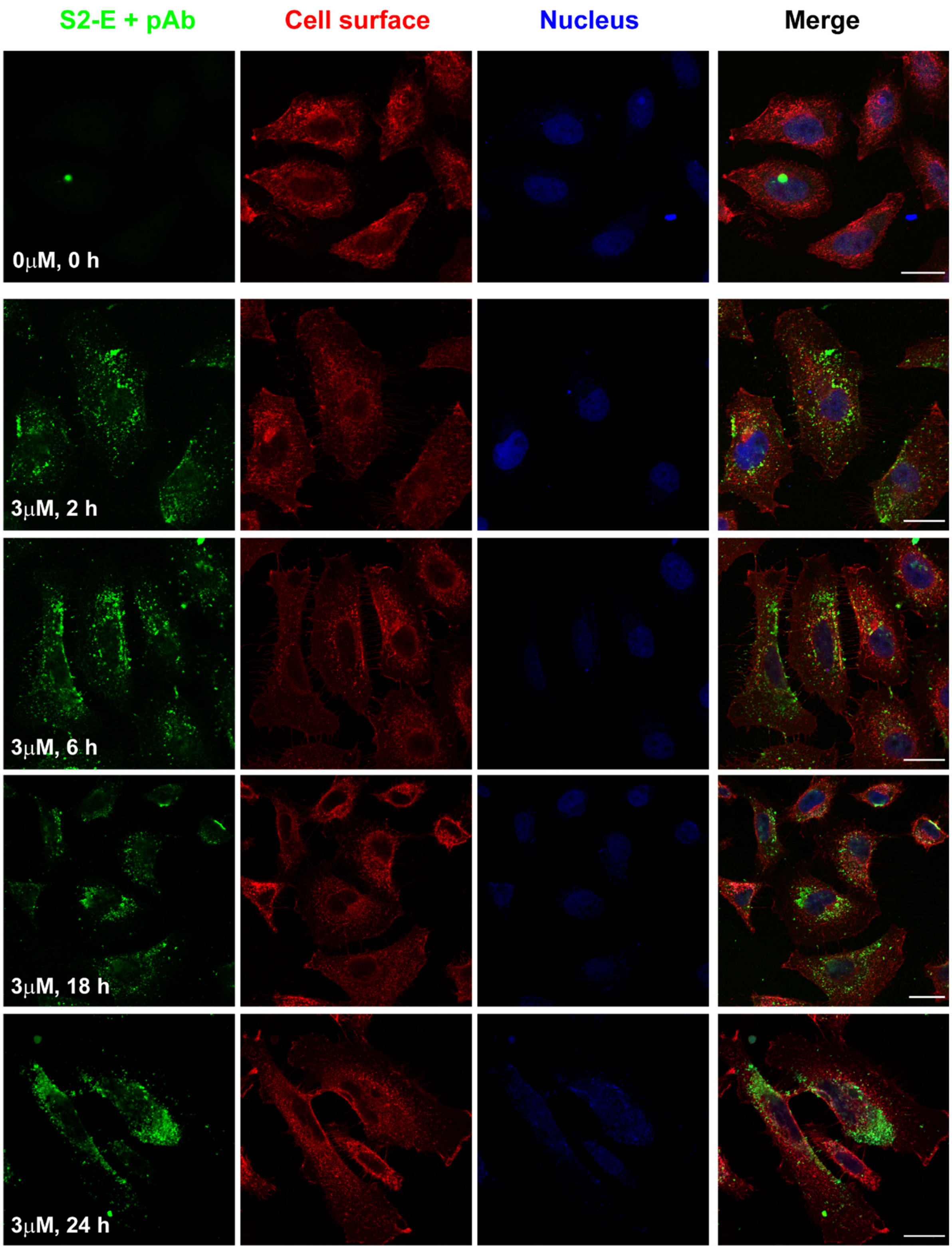

Figure S7. Lower Panel III. Individual channels and composite of upper panels A and J-K

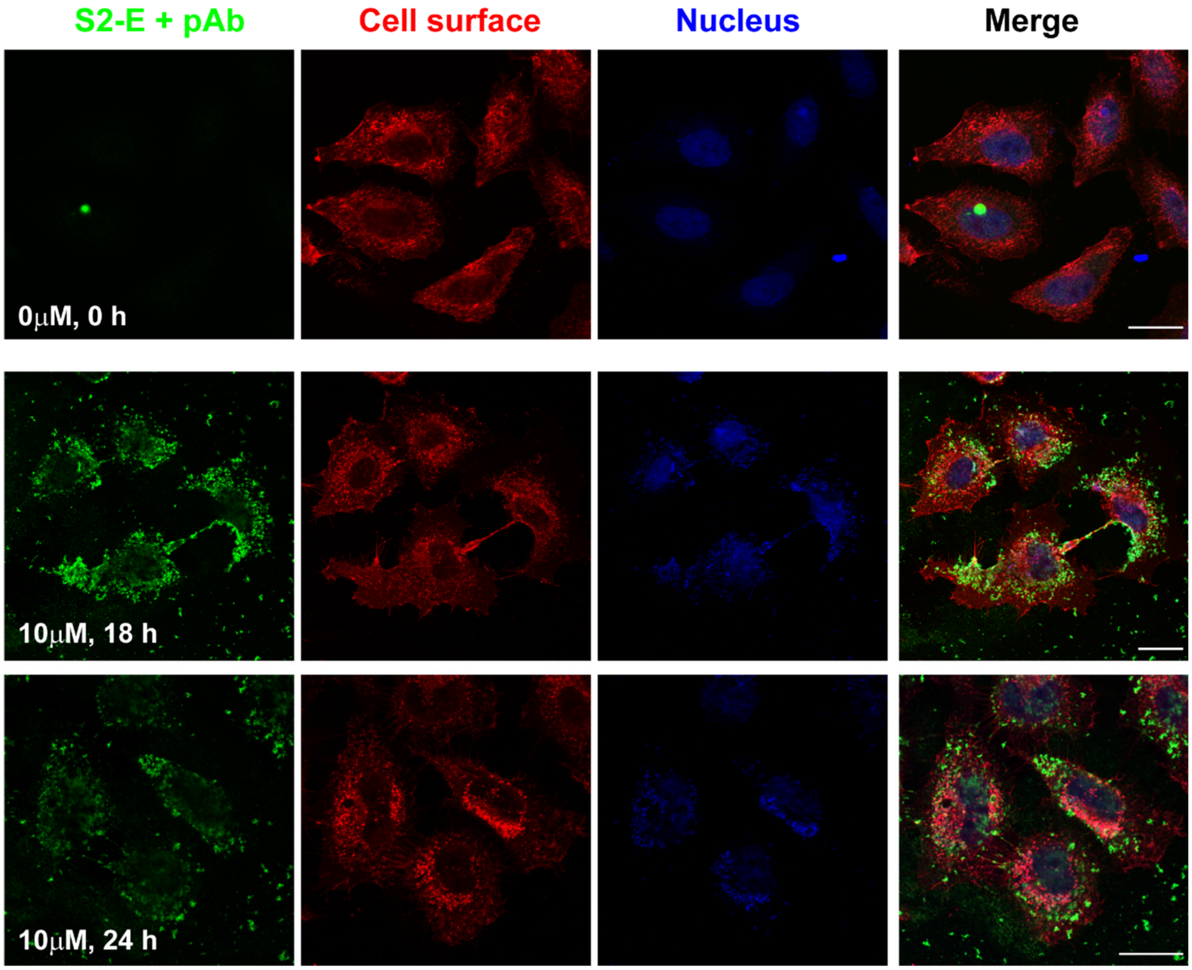

Figure S7 (This page and 3 preceding pages). Unmodified S2-E protein delivered to cells from PMAL-C8 amphipol shows similar cell membrane localization and retrograde kinetics as for the NBD-tagged S2-E protein. The time course and concentration dependency of unmodified S2-E was followed using rabbit anti-E polyclonal antibodies. Rabbit antibodies were detected using anti-rabbit conjugated AF488 (green). Hela cell surface plasma membranes (red) were detected using WGA-AF555, while cell nuclei (blue) were labeled using DRAQ5. Applied unlabeled S2-E concentrations were 1 μM for panels B-E, 3μM for panels F-I, and 10μM in panels J and K. Time points are: 0h and untreated sample (A); for the 1μM series: 2h (B), 6h (C) 18h (D) and 24h (E); for 3μM series: 2h (F), 6h (G) 18h (H) and 24h (I); for cells treated with 10μM unlabeled E: 18h (D) and 24h (E). At 10μM, S2-E observation of cell debris suggest cellular toxicity. Overall, delivery and retrograde transport of unmodified S2-E follows a similar time course as for NBD-labeled S2-E; however, the control sample without added E but treated with anti-E (panel A) did exhibit a weak unspecific binding on the green channel (see also Fig. S5 panel E). The lower panels I (2nd page of Fig.) , II (3rd page), and III (4th page) of the figure show the individual channels of each composite image presented in the upper panel. For comparison purposes, we re-used the untreated control (panel A) at each S2-E concentration 1 μM, 3 μM and 10 μM. For lower panel I (A-D), lower panel II (A, G-I) and panel III (A, J and K). All scale bars are 25 μm. This experiment was independently repeated twice. For further experimental details see the above Supporting Materials and Methods.

Figure S8

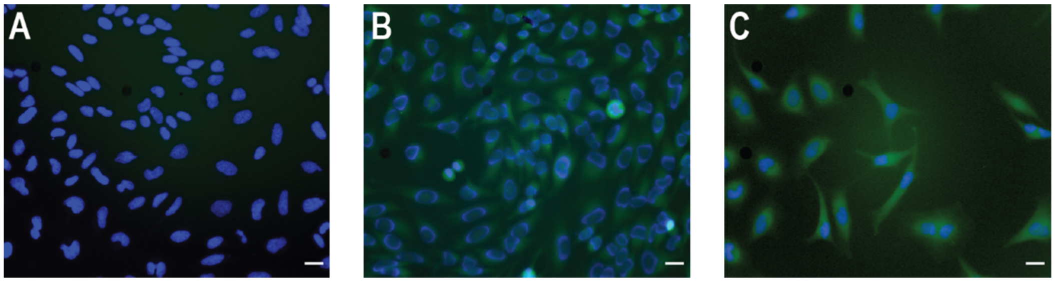

Figure S8. Cysteine-NBD uniformly stains membranes in HeLa cells. A) HeLa cells stained with only NucBlue. **B)** Hela cells stained with NucBlue and 5 µM cysteine-NBD for one hour. **C)** Hela cells stained with NucBlue and 5 µM cysteine-NBD after 18 hours. Note that cysteine-NBD stains all membranes in HeLa cells and this does not occur with S2-E-NBD, even after 18 hours. This indicates that S2-E-NBD is not degraded to the point of liberating NBD dye. This experiment was repeated twice. Scale bars 25 µm.

Figure S9. (2 Page Figure)

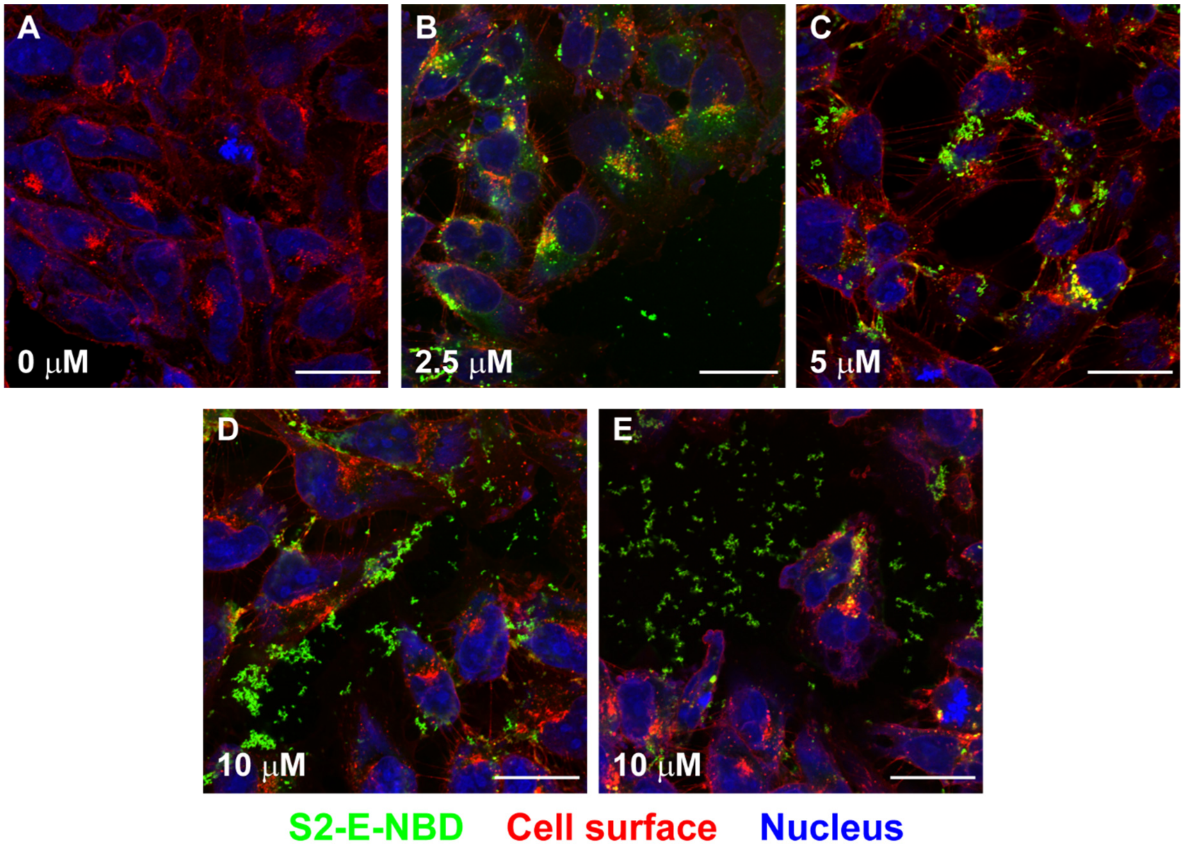

Figure S9. Lower Panel. Individual channels and composite of upper panels A-D. (Figure Page 2 of 2)

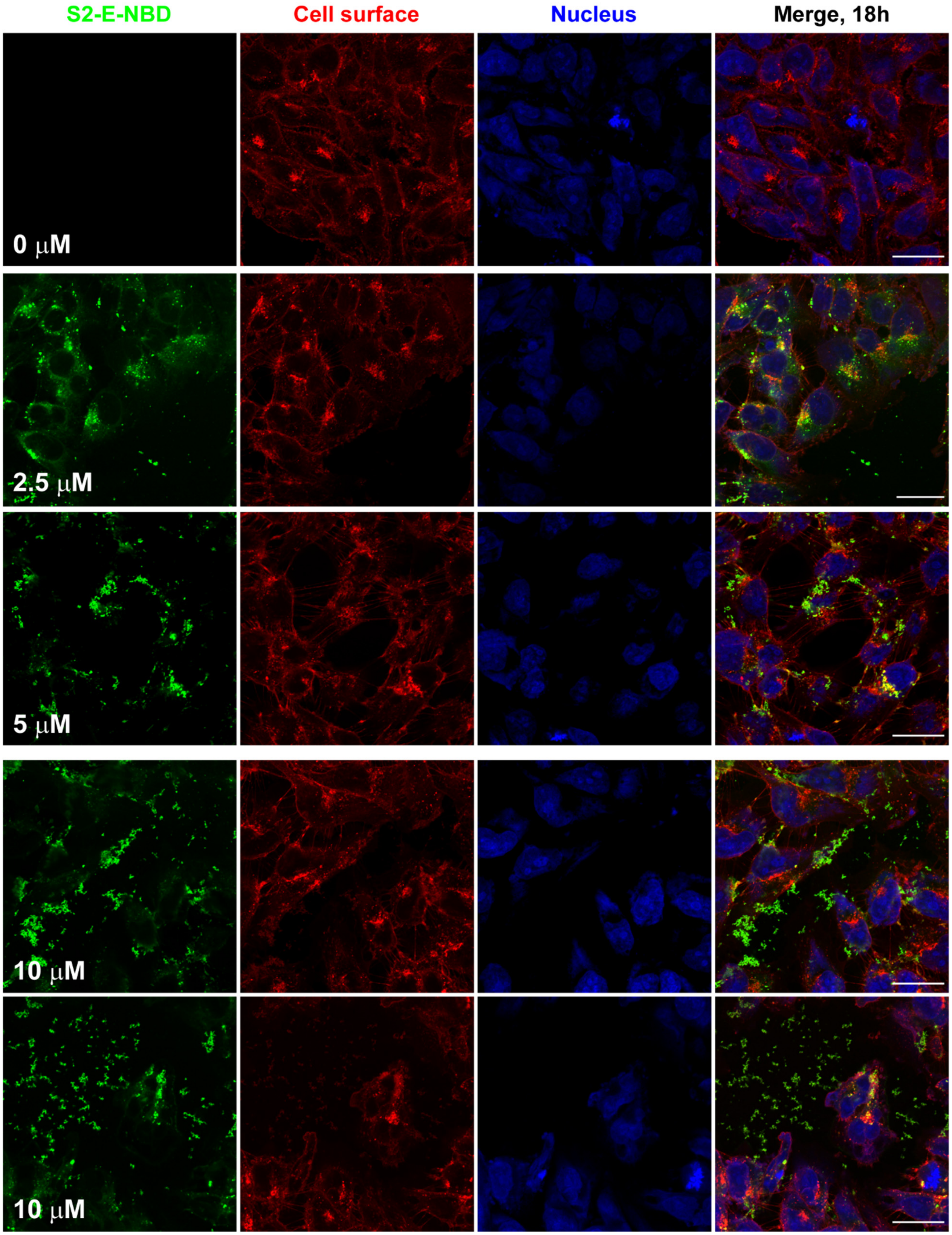

Figure S9 (above 2 pages). Cell membrane delivery and uptake of the SARS-CoV-2 envelope protein with PMAL-C8 amphipol in a human alveolar cell line. Representative confocal microscopy images showing SW1573 cells treated with increasing concentrations of added NBD-labeled S2-E. All time points were collected at 18h. **(A)** untreated control 0µM, **(B)** 2.5µM S2-E in the cell culture, **(C)** 5µM, and 10µM **(D and E)**. Color scheme is: green, NBD-labeled S2-E; red, cell plasma membrane (WGA-AF555); blue, cell nuclei (DRAQ5). As in HeLa cells, at 18hrs after cell treatment the S2-E-NBD signal is primarily at a perinuclear location (panels B and C). For cells incubated with the highest level (10µM) of S2-E-NBD, some of the protein appears to be aggregated (D) while in panel E—top left side, SW1573 shows signs of co-localization of S2-E with the plasma membrane label, indicating cell membrane fragments, possibly suggesting cellular toxicity at 10µM S2-E-NBD. The lower panel (2nd page) of the figure shows the individual channels of each composite image presented in the upper panel. Scale bars are 25 µm. This experiment was conducted once. For further experimental details see the Supporting Materials and Methods.

Figure S10 (2 Page Figure)

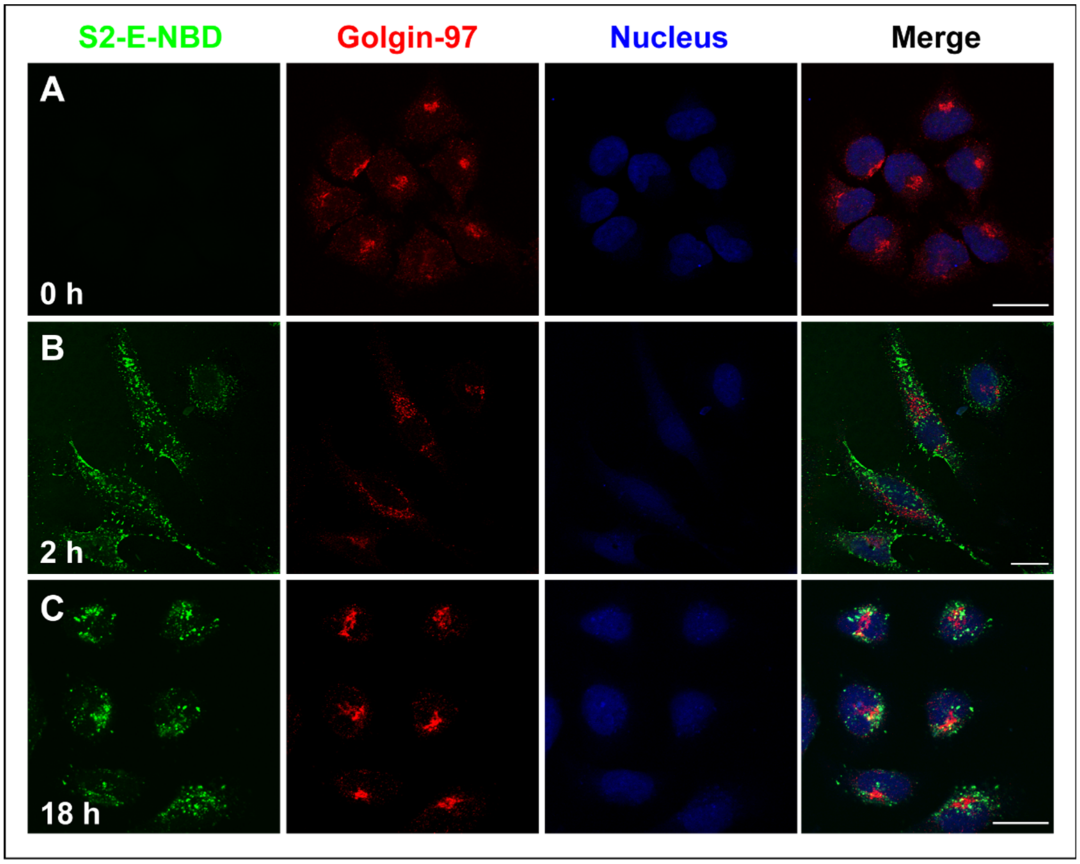

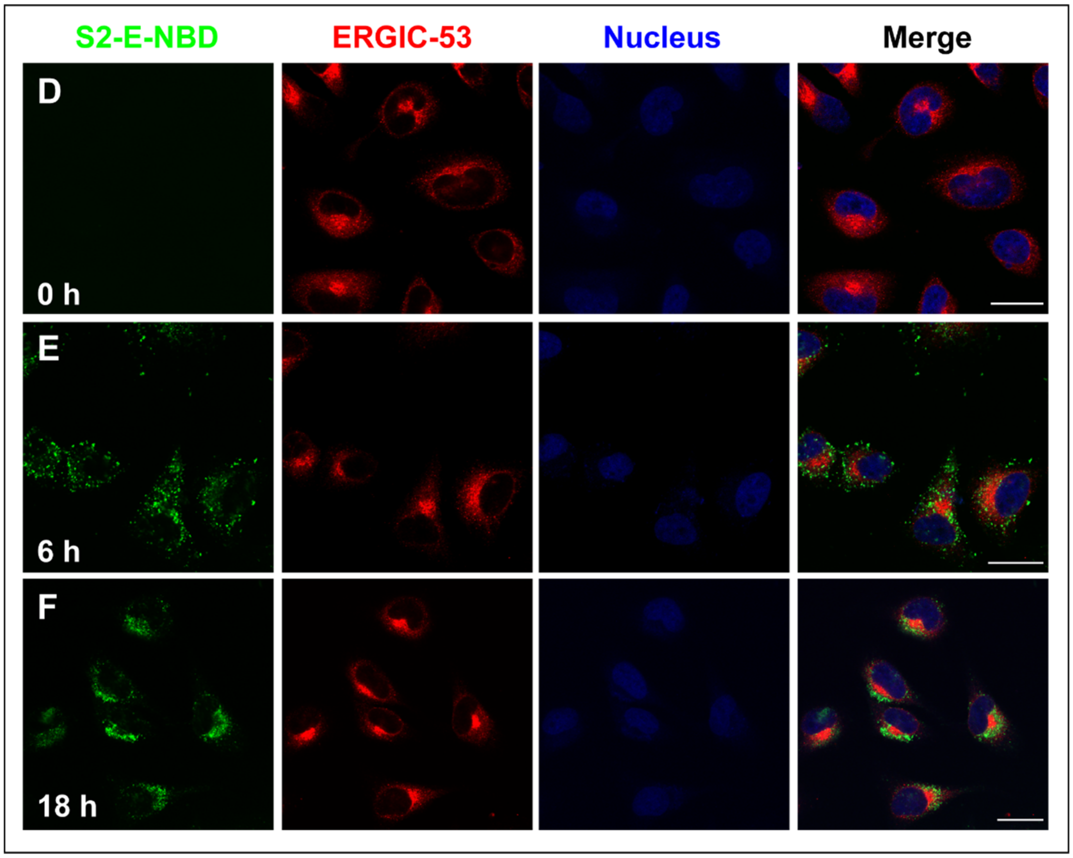

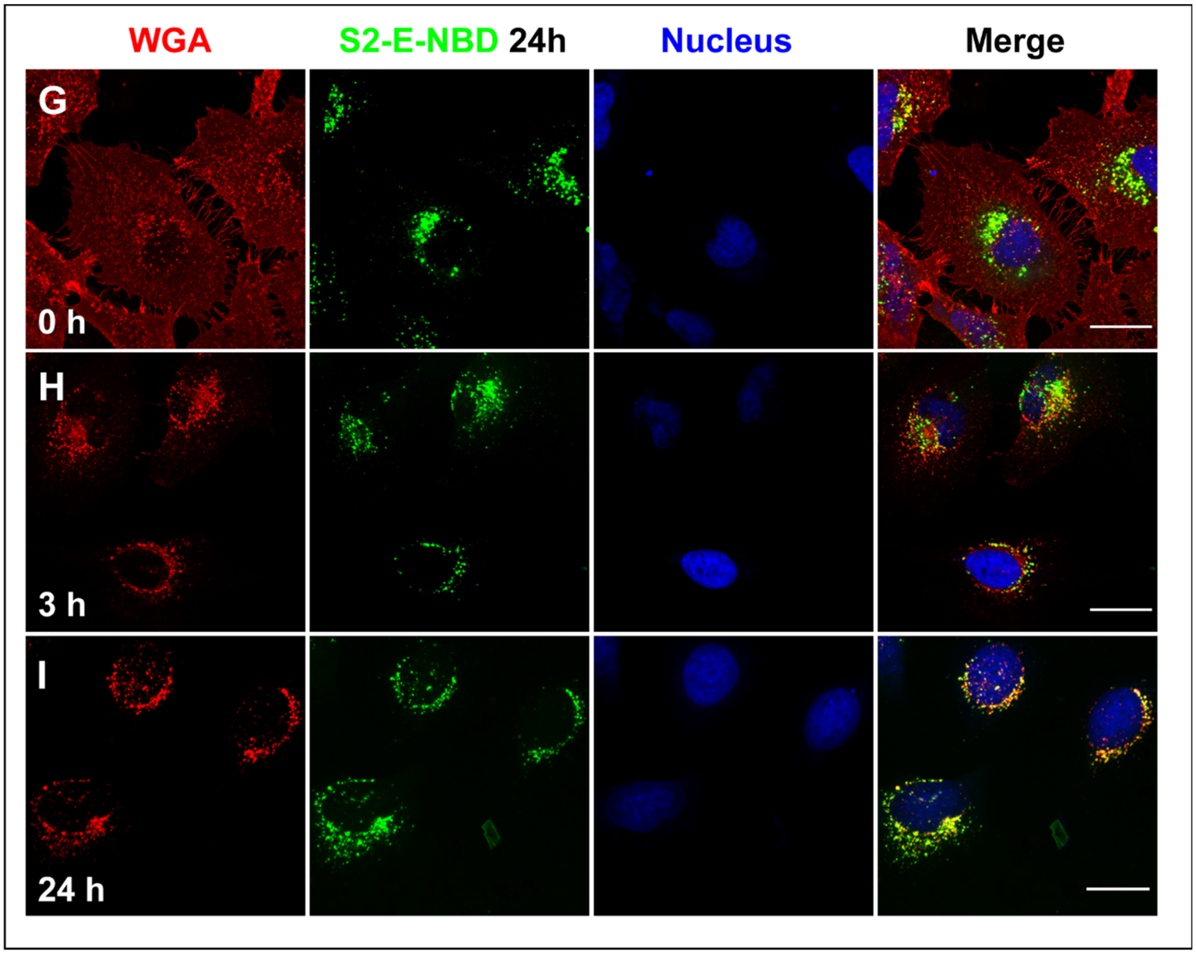

Fig S10. Individual channels for merged images from Figure 3. (above 2 pages) Rows **A** to **C**; HeLa cells treated with 2.5µM E-NBD (green), for 0, 2 and 18 h were tested for Golgi localization (red) using anti-Golgin-97 (Invitrogen cat# A21270 diluted 1:500). Golgin-97 was detected using anti-mouse conjugated AlexaFluor546. Cellular nuclei (blue) were detected using DRAQ5. Notice how the S2-E-NBD signal (green) moves towards the perinuclear space over time; and poorly overlap with the Golgin-97 signal. Correlation analysis using ImageJ plugin Colocalization Threshold for row C: 18h S2-E-NBD (green) versus Golgin-97 (red) yields poor Pearson correlation coefficient. Analysis of several other S2-E-NBD 16-21 h and Golgin-97 confirm further poor correlation (~ 0-0.2). Rows **D** to **F**; HeLa cells treated with 2.5µM S2-E-NBD (green) for 0, 6 and 18 h were tested for ERGIC localization (red) using monoclonal antibody against ERGIC-53 protein. Anti -ERGIC-53 antibodies and cell nuclei were detected using the same conditions used for Golgin-97 in panels A-C. Notice how the S2-E-NBD signal (green) moves toward the perinuclear space over time and appears to overlap with part of the ERGIC-53 signal. Correlation analysis using ImageJ Colocalization Threshold for row F: 18h S2-E-NBD (green) versus ERGIC-53 (red) yields poor Pearson correlation coefficients. Analysis of several other E-NBD 16-24 h and ERGIC-53 confirm further this poor correlation (~ 0-0.2). Rows **G** to **I**; HeLa cells treated with 2.5µM S2-E-AF488 (green) for 24 h were tested for wheat germ agglutinin co-localization (red) using WGA-AF555 at 10µg/mL for the indicates times. In panel G, WGA was added after cell fixation marking the cell membrane, while in panel H, WGA was added 3 h before cell fixation. In panel I, S2-E-AF488 and WGA-AF555 were added together 24 h before cell fixation. Notice how the WGA signal (red) moves towards the perinuclear space over time and overlaps well with the S2-E-AF488 in the merged image (panel I). Correlation analysis using ImageJ Colocalization

Threshold between row I: 24 h S2-E-AF488 (green) versus 24h WGA-AF555 (red) yields a strong Pearson correlation coefficient; this remains the case with either S2-E-AF488 or S2-E-NBD. Analysis of several other equally 24h treated S2-E-AF488 or S2-E-NBD and 24h WGA-AF555 confirm the strong correlation (3 experiments, 2 with S2-E-NBD and one with S2-E-AF488; analyzed cells = 13, 19, 12, for Pearson correlation coefficient averages with standard deviations for 5 images of each experiment were: 0.67 ± 0.15; 0.73 ± 0.16; 0.68 ± 0.097. Scale bars are 25 µm. For further details see online Materials and Methods.

Figure S11

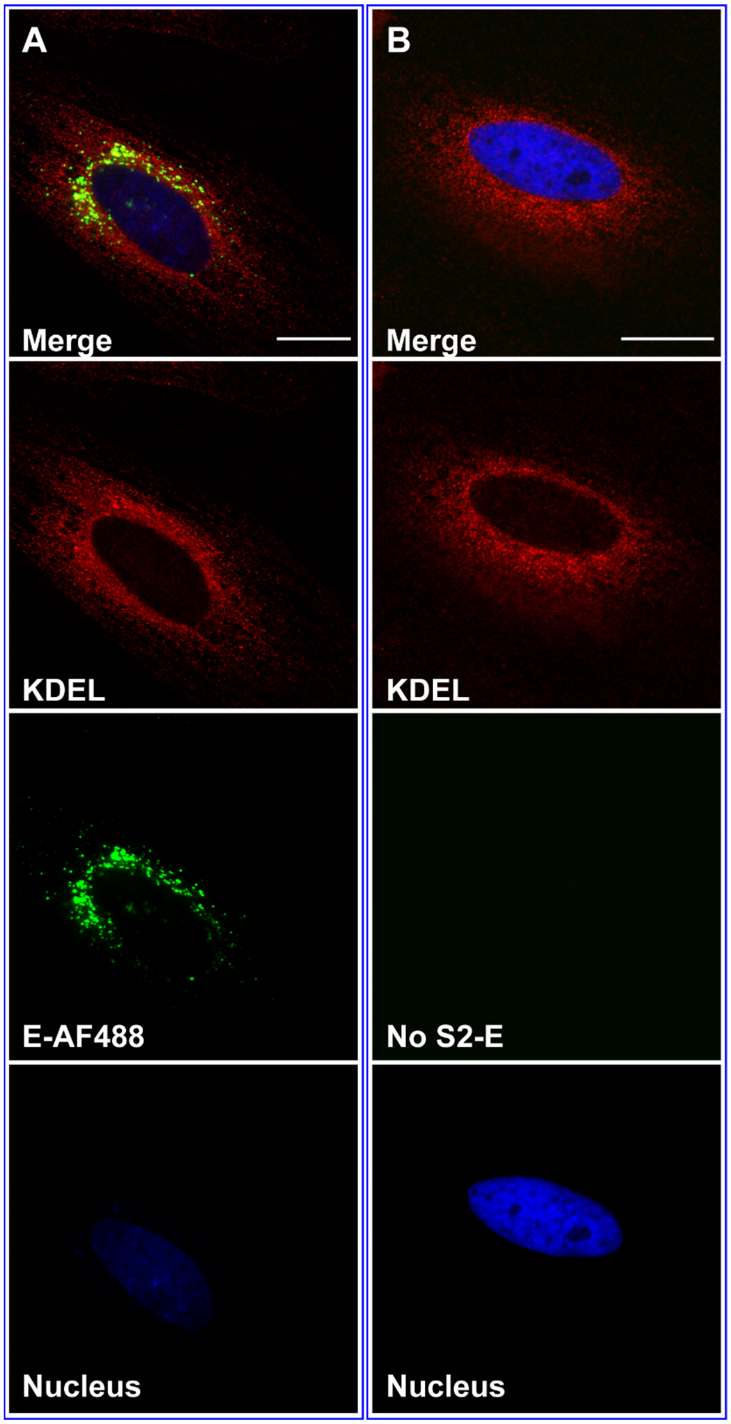

Fig. S11. SARS-CoV-2 envelope protein traffics to the perinuclear space and does not accumulate in the endoplasmic reticulum. Representative confocal microscopy images showing HeLa cells treated for 24 h with 2.5 µM S2-E labeled with AF488 (**A**) or without S2-E (**B**), and probed with the KDEL antibody conjugated to AF555 (Abcam, cat# EPR12668), a known marker for the endoplasmic reticulum. For each column, the first image is the Merge of the below 3 channels, in red anti-KDEL, in green for A, is S2-E-AF488 and in column B no added S2-E, in blue cell nuclei. This experiment was repeated twice once with S2-E-NBD and once with S2-E-AF488. No significant colocalization was observed between KDEL and S2-E-AF488 after 24 h treatment. Scale bars are 20 µm. For more details see online Materials and Methods.

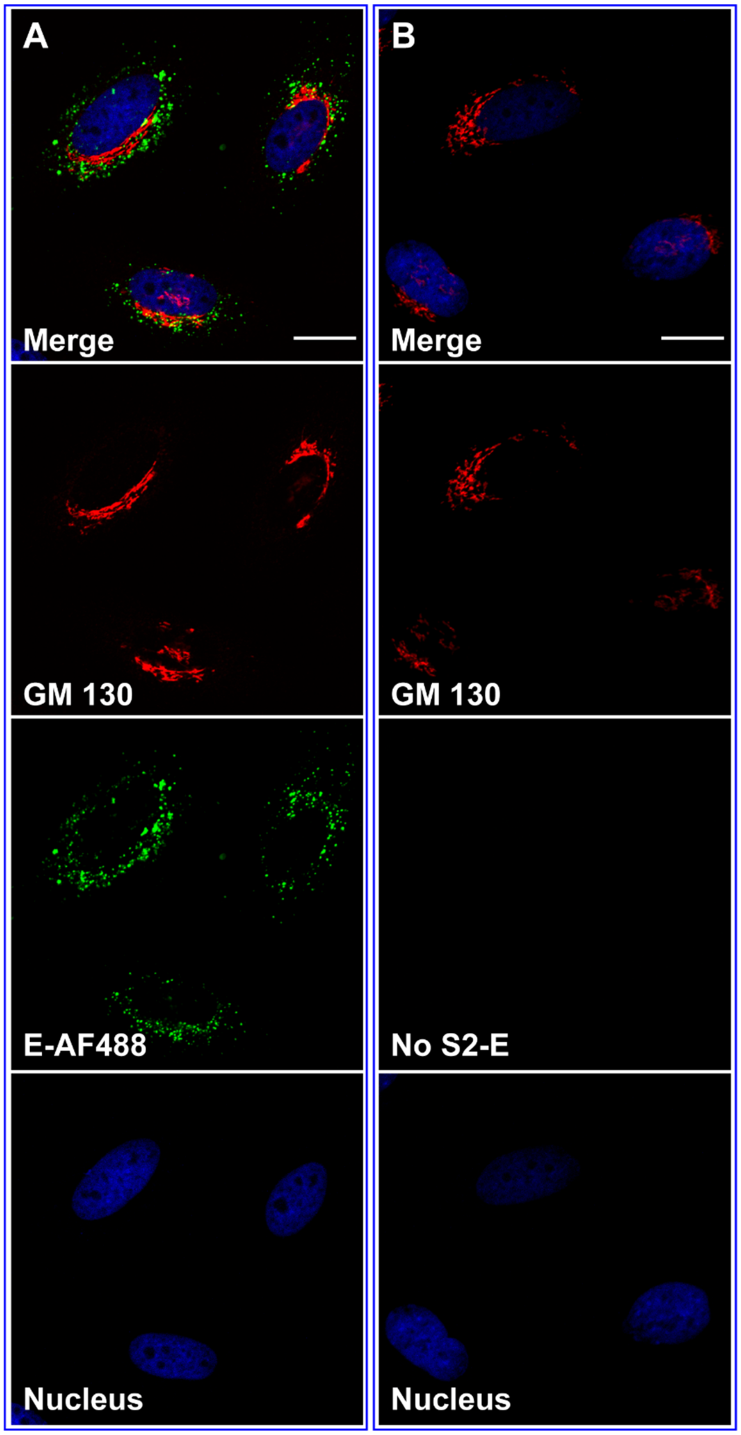

Fig. S12. SARS-CoV-2 envelope protein traffics to the perinuclear space and does not accumulate in the cis-Golgi. Representative confocal microscopy images showing HeLa cells treated for 24h with 2.5 µM S2-E labeled with AF488 (**A**) or without S2-E (**B**) and probed with the GM130 antibody (Cell Signaling, cat #12480S), a known marker for the *cis*-Golgi. For each column, the first image is the Merge of the below 3 channels, red (anti-GM130), green (for A only, S2-E-AF488) and blue (cell nuclei). This experiment was repeated twice, once with S2-E-NBD and once with S2-E-AF488. No significant colocalization was observed between GM130 and S2-E-AF488. Scale bars are 20 µm. For more details see online Materials and Methods.

Figure S13

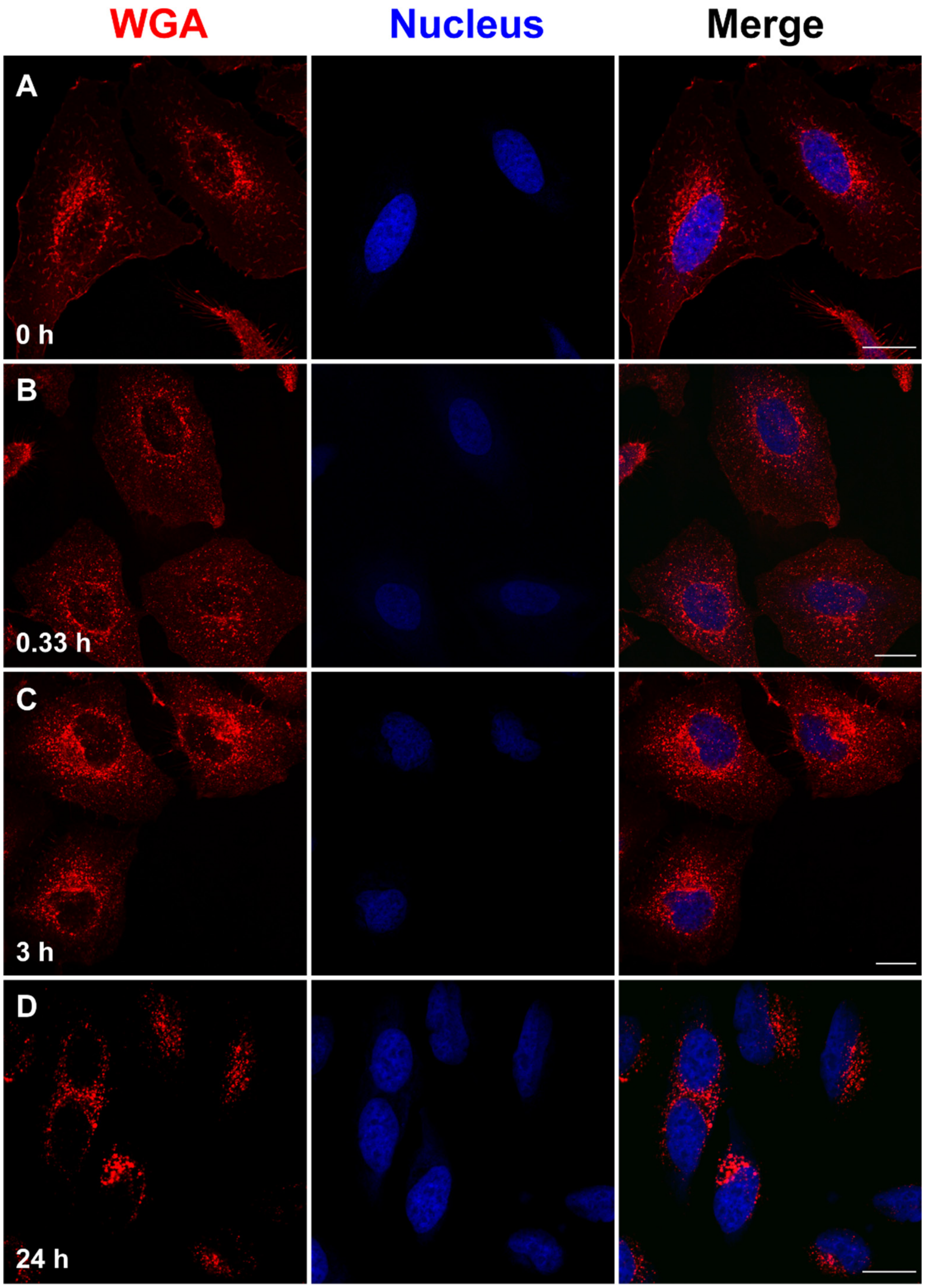

Fig. S13. Wheat germ agglutinin (WGA) protein retrograde traffics and accumulates in the perinuclear space. Representative confocal microscopy images showing HeLa cells treated with 10 µg/mL WGA conjugated to AlexaFluor-555. For each row, the first image in red is WGA-AF555, the second show the blue cell nuclei, and the third is the merge image of both channels. In row **A** cell were WGA-AF555 treated post fixation or 0 h, while in panels B-D cells were treated with WGA and fixed after 20 min (**B**), 3 h (**C**), 24 h (**D**). Notice how, over time, WGA accumulates in the perinuclear space. WGA has been shown in prior studies to be quickly internalized and accumulate in the trans-Golgi network (TGN) See supplemental references (6,7). Experiments were independently repeated three times, twice using S2-E-NBD and once with S2-E-AF488. Scale bars are 20 µm. For more details see online Materials and Methods.

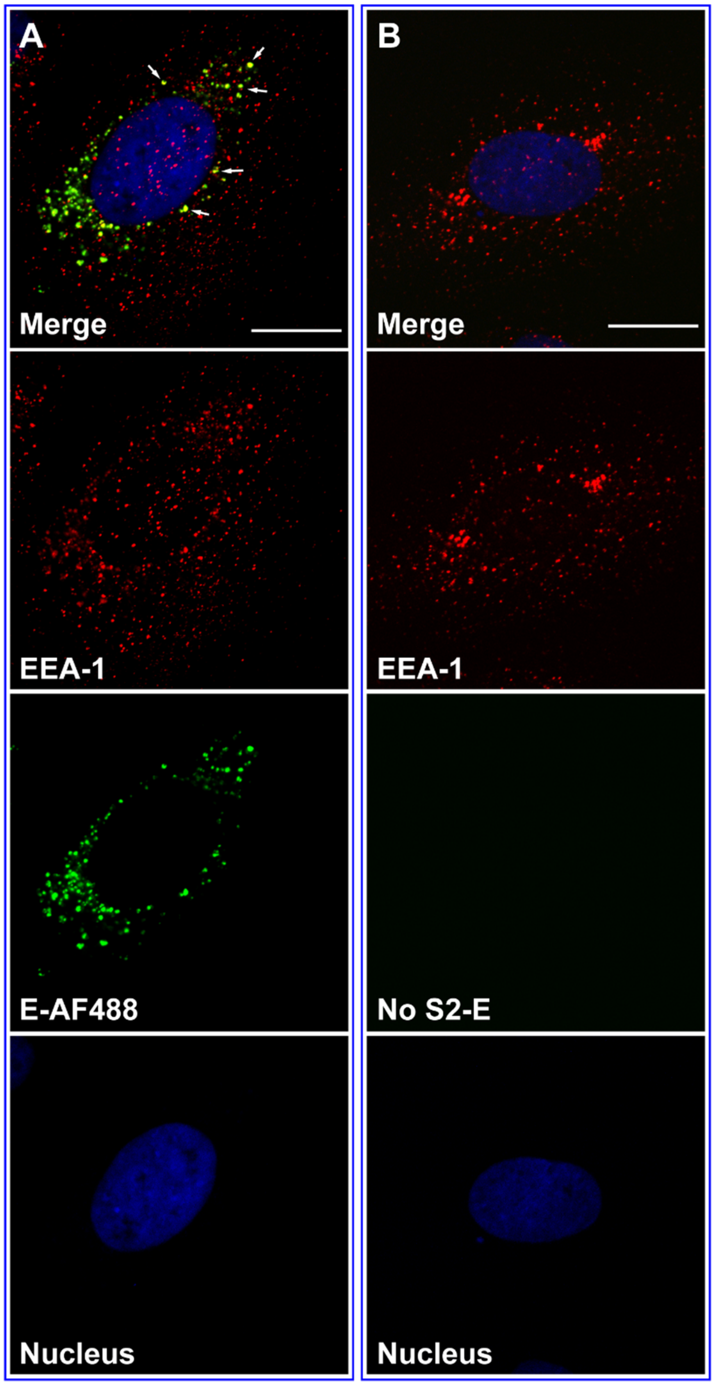

Fig. S14. Some SARS-CoV-2 envelope protein is found in early endosomes at late time points. Representative confocal microscopy images showing HeLa cells treated for 24 h with 2.5 μM S2-E labeled with AF488 (**A**) or without S2-E (**B**) and probed with anti-EEA1 antibody (Invitrogen, cat # MA5-14794), a well establish marker for early endosomes. For each column, the first image is the merge of 3 channels: red (anti-EEA1), green (for A only, S2-E-AF488), and blue (cell nuclei). This experiment was repeated twice, once with S2-E-NBD and once with S2-E-AF488. Some EEA1 vesicles show colocalization with S2-E, most colocalized vesicles appear also as enlarged vesicles. Examples of this are shown with white arrows in panel A merge. Using the image-J plugin colocalization-threshold, the Pearson correlation coefficient between green and red images in column A was determined to be 0.45, suggesting a weak to moderate correlation. Scale bars are 20 μm. For more details see online Materials and Methods.

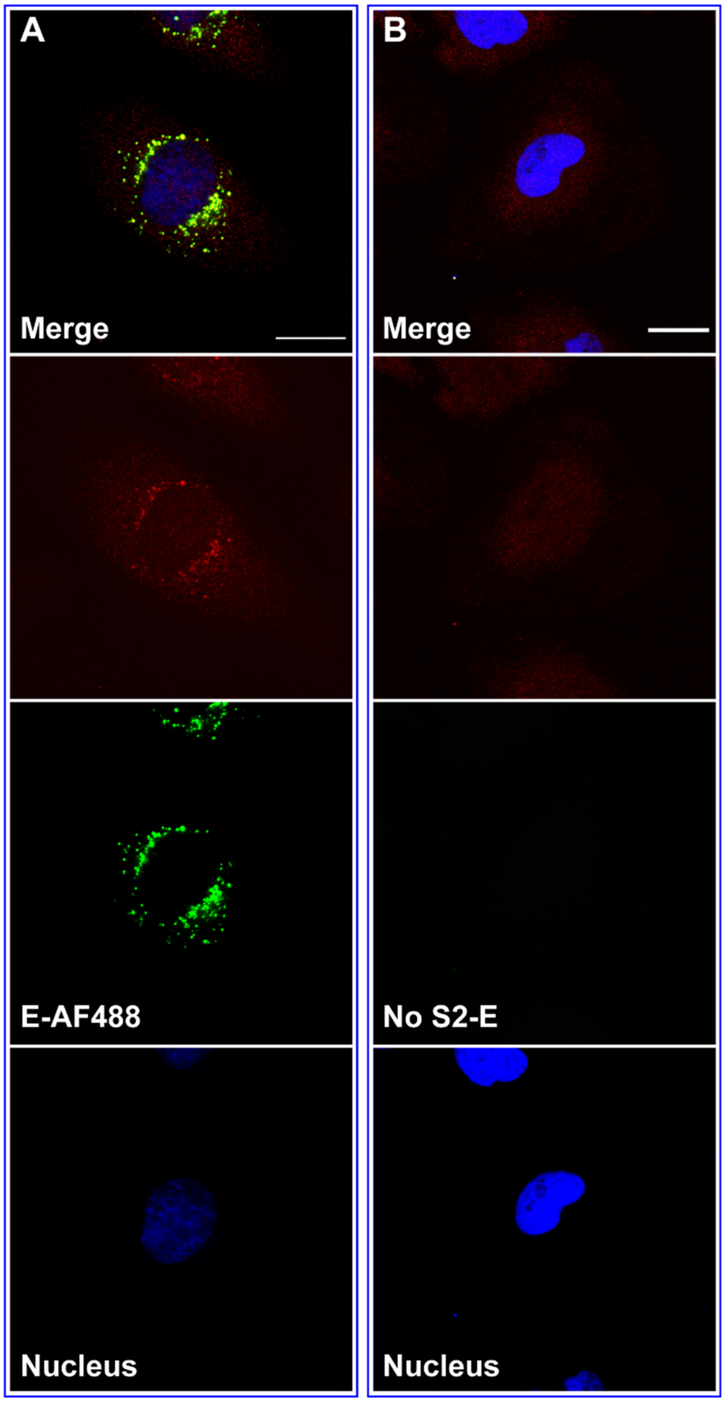

Fig. S15. SARS-CoV-2 envelope protein is also found in late endosomes at late time points. Representative confocal microscopy images showing HeLa cells treated for 24 h with 2.5 µM S2-E labeled with AF488 (**A**) or without S2-E (**B**) and probed with anti-Lamp1 antibody (Encorbio, cat # MCA-5H6), an established marker for late endosomes, endolysosomes, and lysosomes. For each column, the first image is the merge of 3 channels: red (anti-Lamp1), green (for A only, S2-E-AF488) and blue (cell nuclei). This experiment was carried out once. Notice how cells treated with S2-E show enlarged vesicles in the red channel, and how these vesicles overlap with the S2-E-AF488 signal. Using the Image-J plugin colocalization-threshold without ROI, the Pearson correlation coefficient between panel A, green and red images, was determined to be 0.64, suggesting good correlation. Scale bars are 20 µm. For more details see online Materials and Methods.

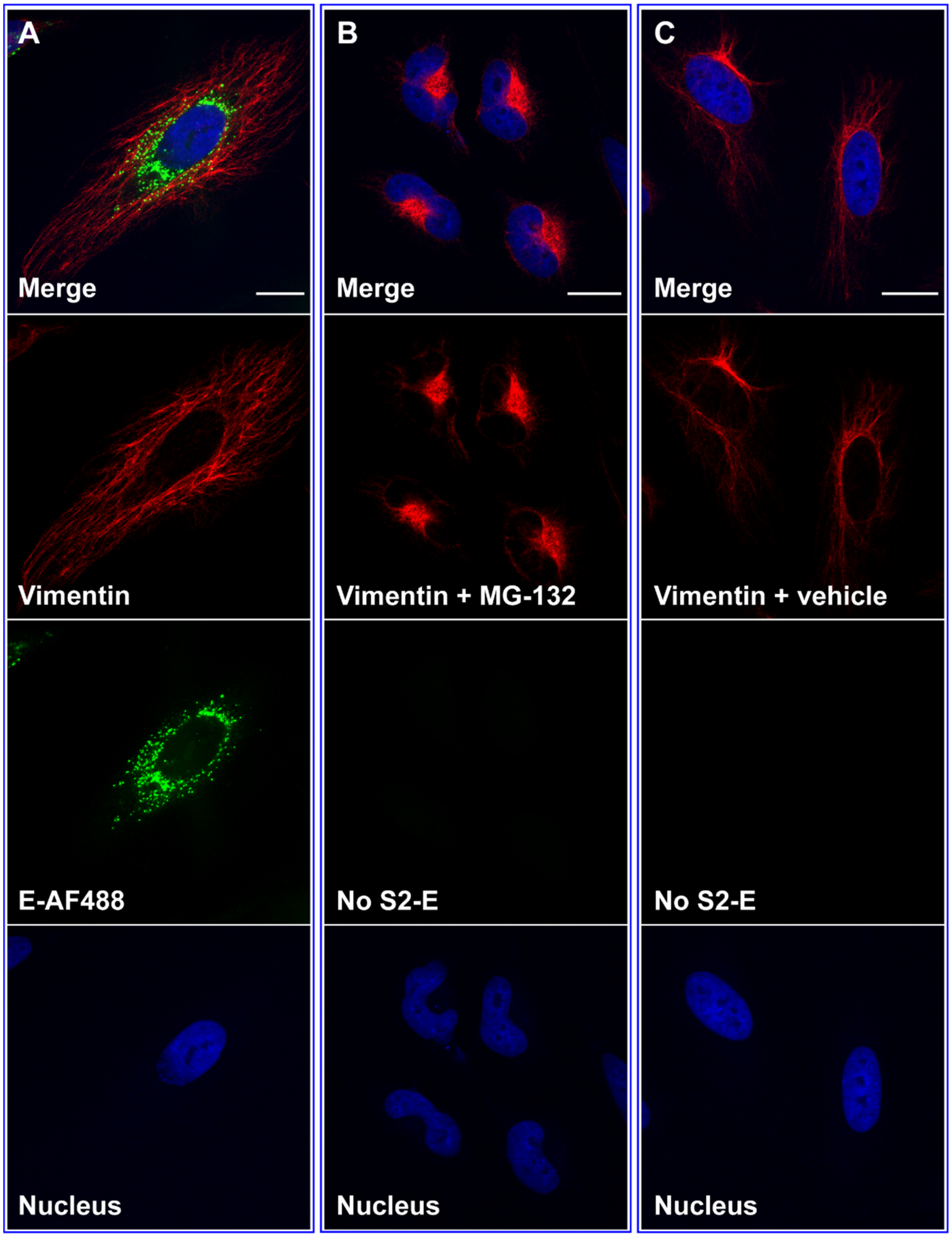

Fig. S16. SARS-CoV-2 envelope protein traffics to the perinuclear space and does not accumulate in aggresome vimentin-cages. Representative confocal microscopy images showing HeLa cells treated for 24 h with 2.5 µM S2-E labeled with AF488 (**A**) and two control samples. As the positive control HeLa cells were treated with 12 µM MG-132 for 21 h (**B**). MG-132 is a protease inhibitor known to induce formation of perinuclear, typically one sided, vimentin cages containing denatured proteins. As a negative control (**C**) show cells treated only with DMSO vehicle. For each column, the first image is the merge of 3 channels: red (anti-vimentin, Cell Signaling, cat #5741S)], green (panel A only, S2-E-AF488), and blue (cell nuclei). This experiment was repeated twice once with S2-E-NBD and once with S2-E-AF488. No vimentin cages were observed, suggesting that the majority of S2-E is not in aggresomes. Scale bars are 20 µm. For more details see online Materials and Methods.
